# Describing variability in pig genes involved in coronavirus infections for a One Health perspective in conservation of animal genetic resources

**DOI:** 10.1038/s41598-021-82956-0

**Published:** 2021-02-09

**Authors:** Samuele Bovo, Giuseppina Schiavo, Anisa Ribani, Valerio J. Utzeri, Valeria Taurisano, Mohamad Ballan, Maria Muñoz, Estefania Alves, Jose P. Araujo, Riccardo Bozzi, Rui Charneca, Federica Di Palma, Ivona Djurkin Kušec, Graham Etherington, Ana I. Fernandez, Fabián García, Juan García-Casco, Danijel Karolyi, Maurizio Gallo, José Manuel Martins, Marie-José Mercat, Yolanda Núñez, Raquel Quintanilla, Čedomir Radović, Violeta Razmaite, Juliette Riquet, Radomir Savić, Martin Škrlep, Graziano Usai, Christoph Zimmer, Cristina Ovilo, Luca Fontanesi

**Affiliations:** 1 Department of Agricultural and Food Sciences, Division of Animal Sciences, University of Bologna, Viale Fanin 46, 40127 Bologna, Italy; 2 Departamento Mejora Genética Animal, Instituto Nacional de Investigación y Tecnología Agraria yAlimentaria (INIA), Crta. de la Coruña, km. 7, 5, 28040 Madrid, Spain; 3 Centro de Investigação de Montanha (CIMO), Instituto Politécnico de Viana do Castelo, Escola Superior Agrária, Refóios do Lima, 4990-706 Ponte de Lima, Portugal; 4 DAGRI – Animal Science Section, University of Florence, Via delle Cascine 5, 50144 Florence, Italy; 5 MED – Mediterranean Institute for Agriculture, Environment and Development, Universidade de Évora, Pólo da Mitra, Apartado 94, 7006-554 Évora, Portugal; 6 Biodiversity School of Biological Sciences, University of East Anglia, Norwich Research Park, Norwich, Norfolk NR47UH UK; 7 Faculty of Agrobiotechnical Sciences Osijek, Josip Juraj Strossmayer University of Osijek, Vladimira Preloga 1, 31000 Osijek, Croatia; 8 Earlham Institute, Norwich Research Park, Colney Lane, Norwich, Norfolk NR47UZ UK; 9 Department of Animal Science, Faculty of Agriculture, University of Zagreb, Svetošimunska c. 25, 10000 Zagreb, Croatia; 10 Associazione Nazionale Allevatori Suini (ANAS), Via Nizza 53, 00198 Rome, Italy; 11 IFIP Institut du porc, La Motte au Vicomte, BP 35104, 35651 Le Rheu Cedex, France; 12 Programa de Genética y Mejora Animal, Institute for Research and Technology in Food and Agriculture (IRTA), Torre Marimon, 08140 Caldes de Montbui, Barcelona, Spain; 13 Department of Pig Breeding and Genetics, Institute for Animal Husbandry, 11080 Belgrade-Zemun, Serbia; 14 Animal Science Institute, Lithuanian University of Health Sciences, Baisogala, Lithuania; 15 Génétique Physiologie et Systèmes d’Elevage (GenPhySE), Université de Toulouse, INRA, Chemin de Borde-Rouge 24, Auzeville Tolosane, 31326 Castanet Tolosan, France; 16 Faculty of Agriculture, University of Belgrade, Nemanjina 6, 11080 Belgrade‐Zemun, Serbia; 17 Kmetijski Inštitut Slovenije, Hacquetova 17, 1000 Ljubljana, Slovenia; 18 AGRIS SARDEGNA, Loc. Bonassai, 07100 Sassari, Italy; 19 Bäuerliche Erzeugergemeinschaft Schwäbisch Hall, Schwäbisch Hall, Germany

**Keywords:** Infectious diseases, Data mining, Next-generation sequencing, Agricultural genetics, Animal breeding, Genetic markers, Genomics, Population genetics

## Abstract

Coronaviruses silently circulate in human and animal populations, causing mild to severe diseases. Therefore, livestock are important components of a “One Health” perspective aimed to control these viral infections. However, at present there is no example that considers pig genetic resources in this context. In this study, we investigated the variability of four genes (*ACE2*, *ANPEP* and *DPP4* encoding for host receptors of the viral spike proteins and *TMPRSS2* encoding for a host proteinase) in 23 European (19 autochthonous and three commercial breeds and one wild boar population) and two Asian *Sus scrofa* populations. A total of 2229 variants were identified in the four candidate genes: 26% of them were not previously described; 29 variants affected the protein sequence and might potentially interact with the infection mechanisms. The results coming from this work are a first step towards a “One Health” perspective that should consider conservation programs of pig genetic resources with twofold objectives: (i) genetic resources could be reservoirs of host gene variability useful to design selection programs to increase resistance to coronaviruses; (ii) the described variability in genes involved in coronavirus infections across many different pig populations might be part of a risk assessment including pig genetic resources.

## Introduction

Coronaviruses (CoVs) are enveloped single-stranded, positive-strand RNA viruses belonging to the Coronaviridae family, which includes four genera (*Alphacoronavirus*, *Betacoronavirus*, *Gammacoronavirus*, and *Deltacoronavirus*). Several viruses of this family constantly and silently circulate or emerge and re-emerge in the human and animal populations causing, in many cases, mild to severe diseases^1–8^. The most recent dramatic example of a novel human coronavirus is the severe acute respiratory syndrome-coronavirus 2 (SARS-CoV-2), detected in the city of Wuhan, China, in December 2019, and that caused the severe pandemic of Coronavirus Disease 2019 (COVID-19) in this Asian country and then worldwide, critically threatening the public health at the global level^9–13^.

Several animal species can act as reservoirs of coronaviruses and different mechanisms have been suggested for host cell and cross-species transmission of coronaviruses infections^14–19^.

Viral entry, that starts from the receptor recognition, is an essential step determining host range and cross-species infection. Coronaviruses encode a spike (S) glycoprotein, which recognizes and binds to the host receptor on the cell surface^20^. The region of the spike protein that mediates the interaction with the host-cell receptor is called receptor-binding domain (RBD). This domain is constituted by the ectodomain subunit S1 which, in turn, has two main domains: the N-terminal domain (S1-NTD) and the C-terminal domain (S1-CTD;^21^). The S1-NTDs are usually responsible for binding sugar components of the receptors^22–25^ whereas the S1-CTDs are responsible for recognizing protein receptors^26–31^. Subsequently, nearby host proteases cleave the spike glycoprotein, which releases the spike fusion peptide S2. The cleaved S2 peptide allows fusion of viral and cellular membranes facilitating virus entry into the host cell^20^. The infection process has two critical and general issues that should be considered: (i) the diversity of the host receptor usage from different coronaviruses and (ii) the different level of sequence similarity of the S1 subunit of the spike from different genera, whereas those from the same genus have significant sequence similarity of this subunit^20^.

A few host receptors, that could be specific or less specific for different coronavirus groups, have been identified: (i) angiotensin-converting enzyme 2 (ACE2) is specific for the alphacoronavirus HcoV-NL63 and the betacoronaviruses SARS-CoV and SARS-CoV-2^32–36^, (ii) aminopeptidase N (APN or ANPEP), described to be the receptor of the human coronavirus NL63 (HcoV-NL63) and other alphacoronaviruses, like the porcine epidemic diarrhea virus or PEDV, the porcine respiratory coronavirus or PRCV and the transmissible gastroenteritis virus or TGEV^25,37,38^ and (iii) dipeptidyl peptidase-4 (DPP4), the receptor of the Middle-East respiratory syndrome coronavirus (MERS-CoV) and a possible receptor for MERS-like bat coronaviruses including the *Tylonycteris* bat coronavirus HKU4 (Bat-CoV HKU4)^39,40^. All these coronavirus receptors also play their own additional physiological functions in the host other than their role in the viral surface recognition step. The most studied host protease for S protein priming is the transmembrane serine protease 2 (TMPRSS2) which is mainly involved in SARS-CoV and SARS-CoV-2 infections^36,41–43^.

Crystal structures resolved for a number of S1 domains of different coronaviruses complexed with their respective receptor, along with functional studies and in silico comparative analyses of receptor sequences across host species, have identified several critical receptor domains and structures that are relevant for the interactions between the host and the infecting viruses^44,45^. These studies also suggested the utilizing capability of receptors from different animal species by coronaviruses, indicating potential cross-species transmission according to the structural compatibility between the spike domains and the host receptors^46,47^.

Structural variations and different expression levels of the receptors and S protein priming proteases could potentially affect the spike/receptor interactions and subsequent spike cleavage efficiency which might cause differences of susceptibility of the host for the coronavirus infection capability and disease progression. A few studies in humans that investigated the *ACE2* and *TMPRSS2* genes reported variants segregating in different cohorts that might confer resistance against SARS-CoV-2 infection or modulate COVID-19 severity^48–53^.

Several coronaviruses (PEDV, PDCV, SADS-CoV and TGEV), that originated from interspecies transmission, infect the pig (*Sus scrofa*) and cause acute gastroenteritis in neonatal piglets and death of the animals, leading to economically relevant problems to the pig industry^7,54^. Genetic resistance to the infection of these coronaviruses might be present within and among pig populations and breeds^55^. Only few studies have evaluated if pigs can become infected with other coronaviruses causing human diseases, such as SARS-CoV or MERS-CoV. These studies challenged the pigs with the two viruses and the obtained results indicated that a small fraction of the challenged animals were SARS-CoV or MERS-CoV antibody positives without any clinical signs or lesions, indicating that, even if remote, transmission of these viruses to the pigs and other animals cannot be excluded^56–58^. Shi et al.^59^ reported that SARS-CoV-2 replicates poorly in pigs but other animals such as ferrets and cats are permissive to infection. Still, Zhou et al.^12^ reported that SARS-CoV-2 could use ACE2 from four animal species including the porcine ACE2 as the receptor to enter the cell in vitro, suggesting that pigs might be potentially susceptible to SARS-CoV-2 infection and could be a potential intermediate host. In other studies, however, pigs did not result to have developed antibodies against SARS-Cov-2 and were negative for viral RNA after intranasal infection^60,61^.

Epidemiological, biological and virological characteristics of coronaviruses, including their demonstrated ability to easily cross species barriers, suggest that pets and livestock should be considered as part of a global control and of a “One Health” approach to evaluate if animals that are close to human contacts could represent a risk source of infections for humans and vice versa^62,63^. Based on the mentioned preliminary evidences on the potential relationships between SARS-CoV-2 and pigs (even if contrasting) and considering (i) the relevance of the pig production systems for meat supply, (ii) that several other coronaviruses circulate in pigs and cause diseases in this livestock species^7,8,40^, (iii) that receptor variants may confer different susceptibility to infections within species^48–53^, iv) that coronaviruses may jump the species barriers easily^5,18,46,57^ and (v) that variability of the RBD region of the spike protein might determine a quite large host spectrum for every coronaviruses^45,64^, as part of a “One Health” approach^63^, it is needed to evaluate the genetic variability segregating in pig populations potentially conferring differences of sensitivity to coronavirus-related diseases.

In this study, we investigated the variability in several pig genes (*ACE2*, *ANPEP*, *DPP4* and *TMPRSS2*) that can serve as receptors or protease for priming the infection of coronaviruses. We also evaluated their relevance in conferring potential differences in susceptibility to coronavirus diseases, also considering a comparative analysis between the corresponding human genes and the information available in other species. Analysis of variability included a total of 22 European pig breeds and wild boars and two Asian pig populations using next generation sequencing data (NGS). This dataset covered a broad number of pig genetic resources raised in Europe^65,66^ in comparison with a few Asian populations. The obtained results could be useful (i) to establish a risk evaluation system in a “One Health” approach, including information on the diversity of pig populations, (ii) to define cross species evolutionary analyses of genes involved in coronavirus infections and (iii) to identify natural genetic variability within the *Sus scrofa* species that could help to design genetic improvement strategies to increase genetic resistance in commercial and autochthonous pig populations against emerging and re-emerging coronavirus diseases.

## Methods

### Identification of polymorphisms by next generation sequencing in different pig populations

#### Animals and whole genome sequencing in DNA pools

Blood samples from pigs were obtained by specialized professionals following standard breeding procedures and health monitoring practices and guidelines at farm or at slaughter. No treatments or other procedures with animals were performed that would demand ethical protocols according to Directive 2010/63/EU (2010) and in compliance with the ARRIVE guidelines. Collected DNA or samples from previous projects were also re-used in this study. This work took advantage from a study design developed within the Horizon 2020 TREASURE project^65–68^. Animals included in the study were 30 or 35 from each of the 22 pig breeds that were investigated. These breeds are raised in nine European countries (from West to East and then North): Portugal (Alentejana and Bísara); Spain (Majorcan Black); France (Basque and Gascon); Italy (autochthonous: Apulo-Calabrese, Casertana, Cinta Senese, Mora Romagnola, Nero Siciliano and Sarda; and commercial breeds: Italian Large White, Italian Landrace and Italian Duroc); Slovenia (Krškopolje pig, hereafter indicated as Krškopolje); Croatia (Black Slavonian and Turopolje); Serbia (Moravka and Swallow-Bellied Mangalitsa); Germany (Schwäbisch-Hällisches Schwein); and Lithuania (Lithuanian indigenous wattle and Lithuanian White old type). Selection of individuals for sampling was performed by avoiding highly related animals (no full- or half-sibs), balancing between sexes, and prioritizing adult individuals or at least animals with adult morphology. All animals were registered to their respective Herd Books. In addition, 35 Italian wild boars, previously genotyped for the absence of introgressed domestic alleles at major loci^69^, were used in this study. Details on the analysed animals and investigated breeds and wild boars, including geographical distribution, are reported in Supplementary Table S1.

For each pig, genomic DNA was extracted from 8–15 mL of peripheral blood (collected in Vacutainer tubes containing 10% 0.5 M EDTA) using either a standardized phenol–chloroform^70^ or the NucleoSpin Tissue commercial kit (Macherey–Nagel, Düren, Germany). A total of 22 DNA pools were constructed from the European pig breeds and one DNA pool was constructed from European wild boars, including in each pool 30 or 35 individual DNA samples pooled at equimolar concentration (Supplementary Table S1). For the 22 DNA pools of the pig breeds, a sequencing library was generated for each DNA pool by using the Truseq Nano DNA HT Sample preparation Kit (Illumina, CA, USA), following the manufacturer's recommendations. Briefly, DNA was randomly sheared to obtain 350 bp fragments which were end polished, A-tailed, and ligated with the full-length adapter for Illumina sequencing with further PCR amplification. PCR products were purified (AMPure XP system) and libraries were analysed for size distribution by Agilent 2100 Bioanalyzer and quantified using real-time PCR. The qualified libraries were then fed into an Illumina HiSeq X Ten sequencer for paired-end sequencing, obtaining 150 bp length reads. The wild boar DNA pool was sequenced from 250 bp fragment libraries, with 100 bp long paired-end reads, on the BGISeq 500 platform, following the provider’s procedures.

#### Quality controls, sequence alignment and variant detection from sequencing data

Reads that were obtained from the sequenced libraries were cleaned by removing adapter sequences and filtering out sequences presenting more than 10% unknown bases (N) and/or containing low quality bases (Q ≤ 5) over 50% of the total sequenced bases. These procedures on FASTQ files were sub-sequentially carried out using FASTQC v.0.11.7 (https://www.bioinformatics.babraham.ac.uk/projects/fastqc/). Then, filtered high quality reads were mapped on the latest version of the *Sus scrofa* reference genome (Sscrofa11.1) using the BWA-MEM algorithm v.0.7.17^71^ and the parameters for paired-end data. Picard v.2.1.1 (https://broadinstitute.github.io/picard/) was used to remove duplicated reads. A summary of whole genome sequencing data statistics is reported in Supplementary Table S2.

Detection of variants on aligned reads was carried out using CRISP v.122713^72^. CRISP parameters were tuned to maximize the discovery of variations (–ctpval -0.6 –minc 1 –EM 0). A three-step filtering procedure was adopted to retain high quality variants:

- first step: (i) retention of only bi-allelic variants, (ii) a minimum read depth (RD_min_) in each pool equal to ten, (iii) a minimum number of alternative reads, over DNA pools, equal to three, (iv) a maximum read depth (RD_max_), in each pool, equal to 68 (computed as proposed by Li^73^; RD_max_ = RD_mean_ + 4√RD_mean_, where RD_mean_ = 42), and (v) removal of variants mapping in low-quality regions or suffering of strand-bias;
- second step: implementation of the quality filter procedures described by Anand et al.^74^. Despite the low false positive rate of CRISP^72^, these procedures allow the filtering out of other possible false variants. In this step, we made use of dbSNP v.150 (^75^; no. of variants equal to 64,535,988). Briefly, variants were initially annotated as reported in dbSNP (“in.dbSNP” class) or not (“novel” class). These two classes were then subdivided in “rare” and “common” variants. Rare variants were defined as variants presenting a minor allele frequency (MAF) lower than 0.0143. This number represents the “ideal” lower limit of detection (i.e. 1/70), since pools were in general composed by 35 diploid individuals (Supplementary Table S1). This is an approximated estimation that did not take into account the average sequencing depth. Then, considering the “rare” class, the Kolmogorov–Smirnov (KS) test was used to compare the distributions of the quality score of the variant of the sub-classes “in.dbSNP” and “novel”. The KS test measures the similarity of the two distributions in a quantitative way via the D-statistics (a metric ranging from 0 to 1). Lower values of *D* indicate more similar distributions. Different cut-off values, in the range 0–50 with steps of 1, were tested. The CRISP quality score (Q_CRISP_) minimizing the *D* value was selected as the best score;
- third step: to globally evaluate the quality of our dataset, the transition-to-transversion ratio (Ts/Tv) was used as quality indicator (1000 Genomes Project Consortium).

Variant detection in the wild boar DNA pool was carried out with Samtools v.1.7^76^ considering a RD_min_ equal to 3.

Polymorphisms were detected in four porcine candidate genes (*ACE2*, *ANPEP*, *DPP4* and *TMPRSS2*) involved in coronavirus infections considering a region spanning 5 kbp upstream and 5 kbp downstream the corresponding gene coordinates as reported in Ensembl database (http://www.ensembl.org/). Information on the annotated features of these genes in the Sscrofa11.1 genome version as retrieved in Ensembl database (release 100, April 2020) are reported in Table 1. Variants were annotated using the Variant Effect Predictor (VEP) v.95.0^77^, by predicting with SIFT v.5.2.2^78^ their impact to the protein function. Variants that affected the protein coding regions were manually checked. Pipelines were developed either in Python v.2.7.12 or in R v.3.4.4^79^; the Kolmogorov–Smirnov test was carried out with the function “*ks.test*”. SNP allele frequencies (AF) were estimated by counting the number of reads covering the SNP position.

**Table 1:** Candidate genes investigated in the present study.

Gene name	Gene symbol	Pig				Human
		SSC location^1^	Gene^2^	Transcript^3^	Protein-Length^4^	Protein^5^
Angiotensin I converting enzyme 2	*ACE2*	X:12099853-12151275:-1	ENSSSCG00000012138	ENSSSCT00000034032.2	K7GLM4-805	Q9BYF1
Alanyl aminopeptidase, membrane	*ANPEP*	7:55351083-55373881:-1	ENSSSCG00000001849	ENSSSCT00000086218.1	A0A5G2QI26(P15145*)-1017	P15144
Dipeptidyl peptidase 4	*DPP4*	15:68660849-68743818:-1	ENSSSCG00000015894	ENSSSCT00000067722.1	A0A5G2Q7G7(P27487*)-833	P27487
Transmembrane serine protease 2	*TMPRSS2*	13:204876561-204902561:-1	ENSSSCG00000024336	ENSSSCT00000041631.2	A0A287AFA0-526	O15393

^1^Porcine chromosome, starting position, ending position, gene orientation. Coordinates are based on the Sscrofa11.1 reference genome; ^2^ Ensembl gene identifier; ^3^ Ensembl canonical transcript identifier (it is defined as the longest CCDS translation with no stop codons); ^4^ UniProtKB accession number related to the Ensembl canonical transcript. The number of residues of the protein is reported; ^5^ UniProtKB accession number.

*Alternative reviewed entry (Swiss-Prot).

### Mining sequence data from other whole genome resequencing datasets in public databases

As European and Asian pigs derives from independent domestication routes (e.g.^80^), for comparative analyses with information obtained from European pig breeds, sequence data of five Chinese Meishan pigs and two Asian wild boars were retrieved from the EMBL-EBI European Nucleotide Archive (ENA) repository (http://www.ebi.ac.uk/ena), project PRJEB9922. Reads were aligned with BWA-MEM and detection of variants was carried out with Samtools, considering a RD_min_ equal to 3. Variants affecting the protein coding regions of the same four candidate genes (*ACE2*, *ANPEP*, *DPP4* and *TMPRSS2*) were manually checked, were annotated using VEP, and their impact was predicted with SIFT v.5.2.2. A summary of whole genome sequencing data statistics is reported in Supplementary Table S2.

### Variants in porcine candidate genes retrieved from Ensembl database

Genome variants affecting the protein coding sequence (i.e. missense, frameshift and stop gain/loss variants) and the related single amino acid polymorphisms (SAPs) of the *ACE2*, *ANPEP*, *DPP4* and *TMPRSS2* porcine genes were downloaded from Ensembl database (release 100, April 2020)^81^, as information annotated against the Sscrofa11.1 reference genome version of *Sus scrofa* and derived from dbSNP. The impact on the protein function was predicted with SIFT v.5.2.2.

### Comparative analysis between pig and human ACE2, ANPEP, DPP4 and TMPRSS2 protein sequences

Sequence identity between the pig and human ACE2, ANPEP, DPP4 and TMPRSS2 proteins was obtained via sequence alignments carried out with Clustal Omega^82^ as implemented in UniProt^83^. Details about genes, transcripts and protein accessions numbers used in this analysis are reported in Table 1. The identification of protein residues functionally relevant for coronavirus disease infections in humans (SARS, MERS and the novel COVID-19) was carried out through a survey of the literature that focused on human ACE2, ANPEP, DPP4 and TMPRSS2 proteins. Our attention was focused on all protein residues either interacting with coronavirus proteins or functional for the biological activity of the selected proteins, including active sites, substrate sites, ions binding sites, residues in interaction patches and glycosylation sites. These protein residues were selected according to 3D structural analyses and related literatures that identified key roles of these sites in the interaction with the virus spike proteins and the functions of the host protein in virus infections (see Supplementary material for details and the extensive references). We analyzed whether the identified residues were conserved in the porcine proteins via protein sequence alignments as reported above.

## Results

### Candidate gene polymorphisms detected in European pig breeds and wild boars

**Figure 1:**
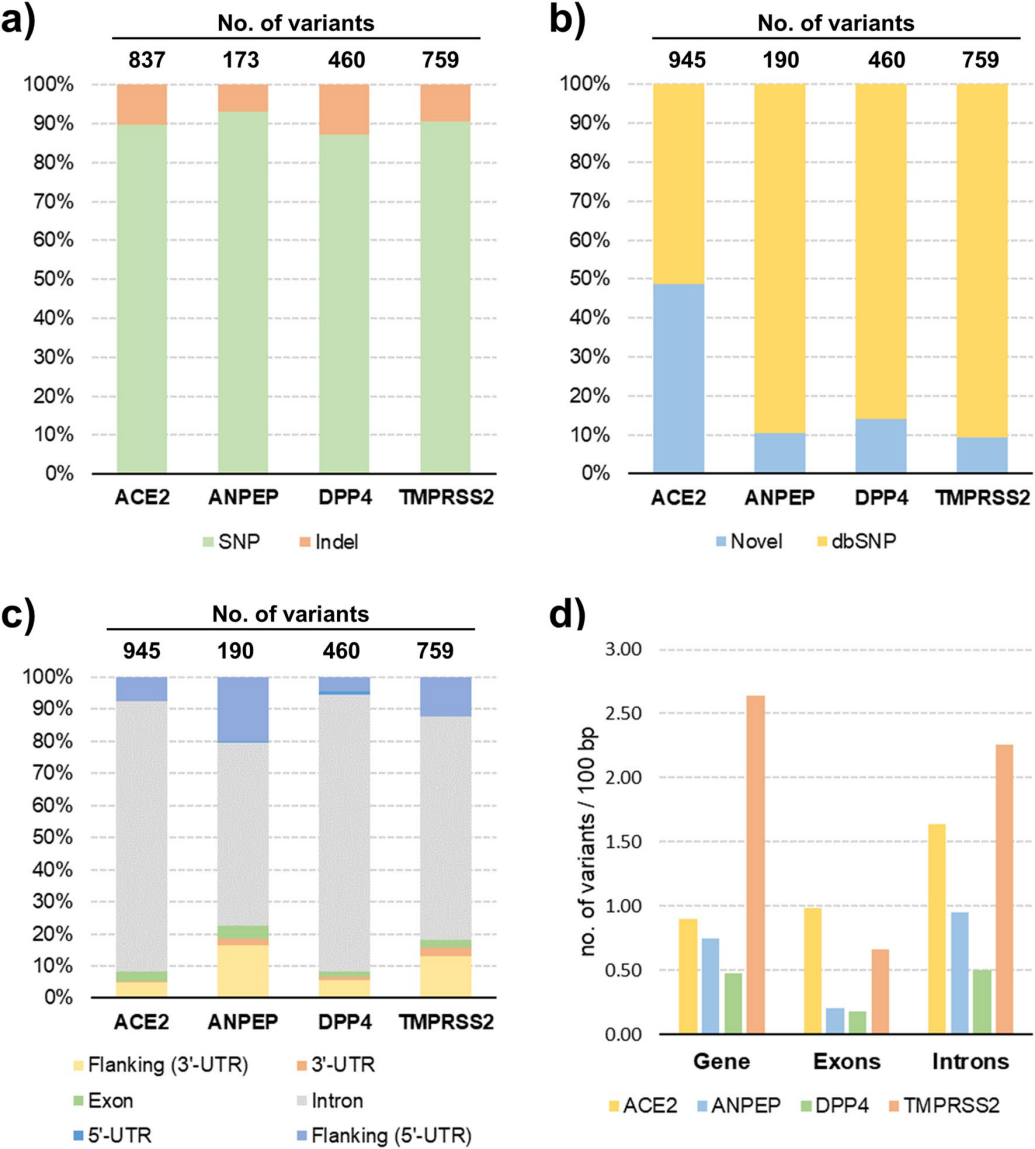
Variants in candidate genes discovered in the analysis of European pig breeds and wild boars. (**a**) Number of called single nucleotide polymorphisms (SNP) and insertions/deletions (indel); (**b**) Classification of variants as novel or already known (deposited in dbSNP); (**c**) Variant location at the gene level (untranslated region: UTR); (**d**) Expected distance of discovered variants stratified by gene feature. Gene length includes UTRs and flanking regions of 5 kbp upstream [flanking (5′-UTR)] and downstream [flanking (3′-UTR)]. Variant counts can differ since variants can co-locate or have multiple consequences as predicted with VEP tool. Details are given in Supplementary Table S3.

We identified a total of 2229 variants (single nucleotide polymorphisms: SNPs; and insertion/deletions: indels) in the four candidate genes and their flanking regions (*ACE2* = 837; *ANPEP* = 173, *DPP4* = 460 and *TMPRSS2* = 759) by mining whole genome resequencing data produced from 22 European pig breeds and European wild boars (Supplementary Table S3). On average, 90% of the detected variants were SNPs and the remaining 10% were indels (Fig. 1a). About 26% of these variants were novel and detected for the first time in this study whereas 74% of the identified polymorphisms were already deposited in dbSNP. *ANPEP*, *DPP4* and *TMPRSS2* genes included a comparable fraction of novel variants (from 9 to 14%) whereas about 50% of the *ACE2* gene variants was novel (Fig. 1b; Supplementary Table S3). We further evaluated the distribution of variants considering different gene features. Overall, the largest proportion of polymorphisms (~ 78%) was within introns whereas variants in the coding regions represented only 3% of the total number of polymorphic sites. Untranslated (UTRs) and flanking regions had a similar number of DNA polymorphisms (~ 9%; Fig. 1c; Supplementary Table S3). Variant density (number of variants/100 bp of gene length) was analysed for all genes and all gene regions. *TMPRSS2* had the highest variant density, considering the total length of the gene, whereas *DPP4* had the lowest density of polymorphic sites (Fig. 1d). *ACE2* had the highest density of variants in the coding regions (about 1 variant every 100 bp).

**Figure 2:**
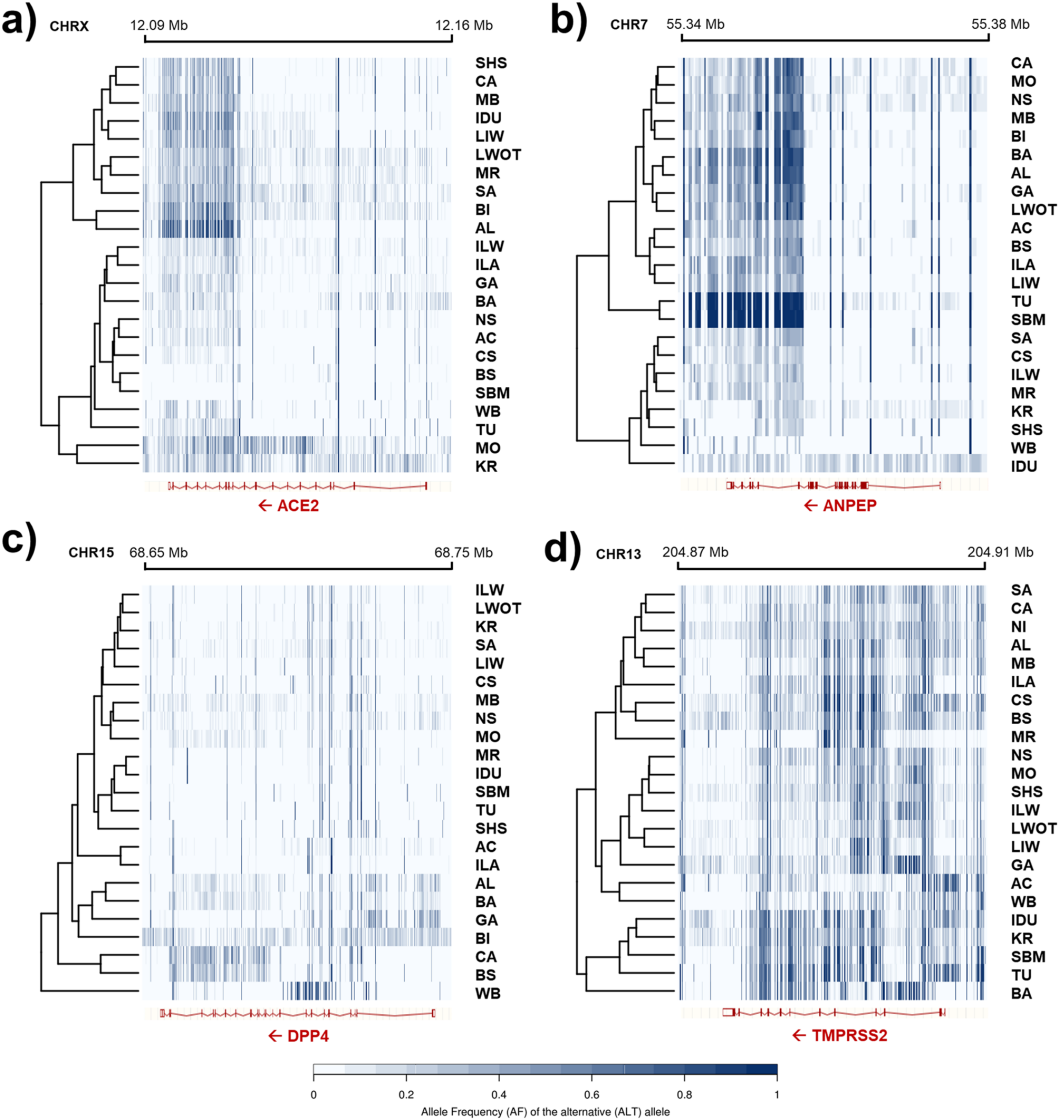
Representations of variant allele frequency values in the analyzed candidate genes plotted for each breed and position, considering the alternative (ALT) allele (defined considering the corresponding nucleotides on Sscrofa11.1 genome version). (**a**) *ACE2*, (**b**) *ANPEP*, (**c**) *DPP4* and (**d**) *TMPRSS2*. Acronyms of the breed name are the following: Alentejana, AL; Apulo-Calabrese, AC; Basque, BA; Bísara, BI; Black Slavonian, BS; Casertana, CA; Cinta Senese, CS; Gascon, GA; Krškopolje, KR; Lithuanian Indigenous Wattle, LIW; Lithuanian White Old Type, LWOT; Majorcan Black, MB; Mora Romagnola, MR; Moravka, MO; Nero Siciliano, NS; Sarda, SA; Schwäbisch-Hällisches Schwein, SHS; Swallow-Bellied Mangalitsa, SBMA; Turopolje, TU; Italian Duroc, IDU; Italian Large White, ILW; Italian Landrace, ILA; Wild Boar, WB.

Allele frequency distribution of the identified variants over the four genes in the 22 pig breeds and wild boars, estimated on the number of reads carrying alternative forms as obtained from the sequenced DNA pools, are reported in Fig. 2.

### Analysis of porcine ACE2, ANPEP, DPP4 and TMPRSS2 deduced protein sequence variants

**Figure 3:**
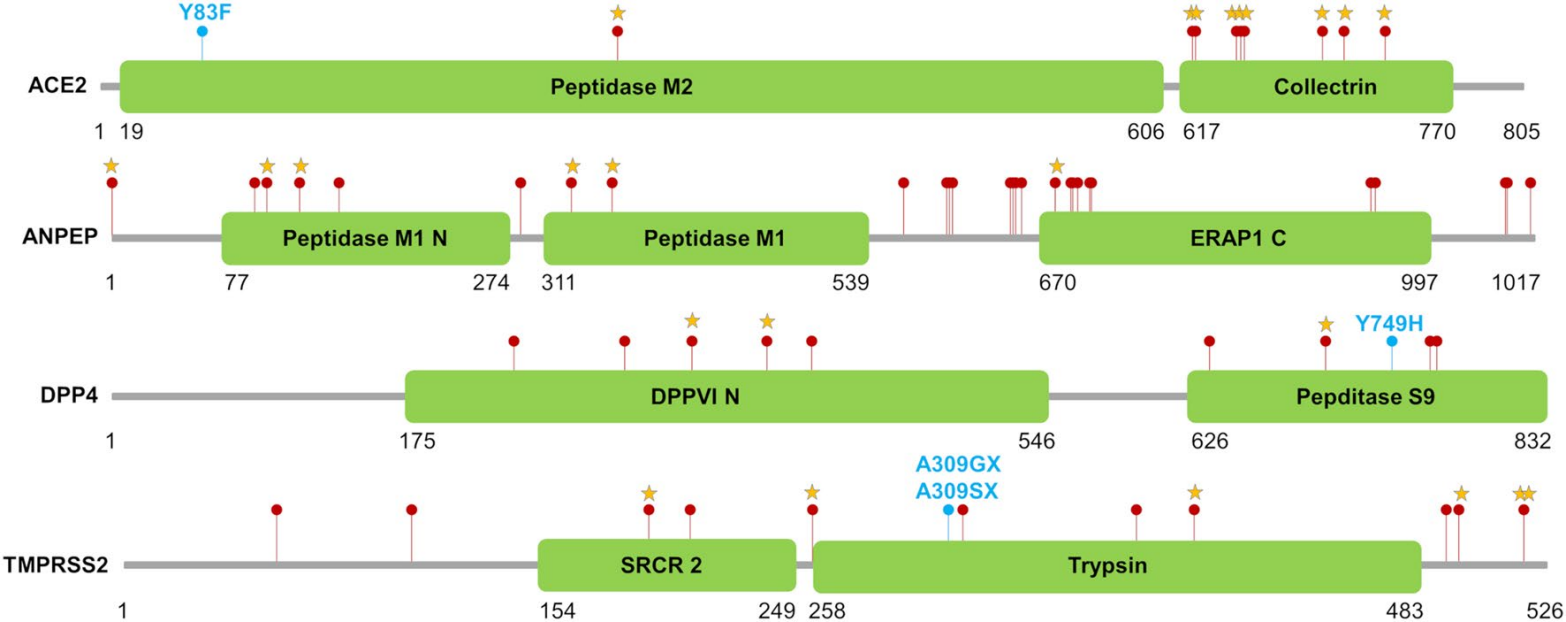
Protein coding variants affecting the ACE2, ANPEP, DPP4 and TMPRSS2 proteins. Red dots indicate the variants retrieved from Ensembl database. Light blue dots and stars indicate novel and known variants identified from the resequencing datasets, respectively. Protein domains and their coordinates are based on the Pfam database (https://pfam.xfam.org/) considering the protein identifiers provided in Table 1.

Protein variants might play important roles in receptor-driven host-virus interactions and in the function of the host proteinases involved in the progression of coronavirus infections. The human ACE2, ANPEP, DPP4 and TMPRSS2 proteins have been extensively studied and several key residues have been identified in the corresponding proteins (see references cited in the Supplementary Material for a complete analysis of the available studies). To infer potential effects of the deduced variants identified using DNA sequencing data in the porcine ACE2, ANPEP, DPP4 and TMPRSS2 translated proteins (constituted by 805, 1017, 833 and 526 residues, respectively), we first compared the pig protein sequences with those of the human homologous proteins. Then, we evaluated the impact of protein coding variants identified in pigs and derived by combining the different datasets explored in this study (DNA pools from European breeds and wild boars; Asian pig genomes; Ensembl database). Figure 3 reports the position of the identified and analysed protein coding variants located in the four encoded proteins.

#### Pig vs human protein sequence comparisons

Overall sequence homology between the pig and human ACE2, ANPEP, DPP4 and TMPRSS2 proteins showed that the two species share 81.7, 74.7, 81.0, 68.5% identical residues, respectively. In these proteins, a total of 82 (ACE2), 19 (ANPEP), 24 (DPP4) and 30 (TMPRSS2) key residues are considered essential either for the virus-host interaction or for the functional activity (Supplementary Table S4). At these key positions, the pig and human proteins showed a total of 62/82 (76%), 14/24 (58%), 21/30 (70%) and 8/8 (100%) identical residues, respectively.

In more details and considering the different functions of the protein positions, the analysis of the ACE2 residues essential for the virus-host interaction showed 25/35 identical residues between the two species (Supplementary Table S4). ANPEP and DPP4 have 3/10 and 8/15 identical residues needed for the virus-host interaction (Supplementary Table S5–S6). The active and binding sites of the four proteins were all conserved across species (13/13 for ACE2, 5/5 for ANPEP, 6/6 for DPP4 and 8/8 for TMPRSS2; Supplementary Table S4–S7). Other sites, such as cleavage, glycosylation and host protein–protein interaction sites showed different degrees of conservation between the human and pig sequences (Supplementary Table S4–S7).

#### Protein coding variants deduced from whole genome resequencing datasets

**Figure 4:**
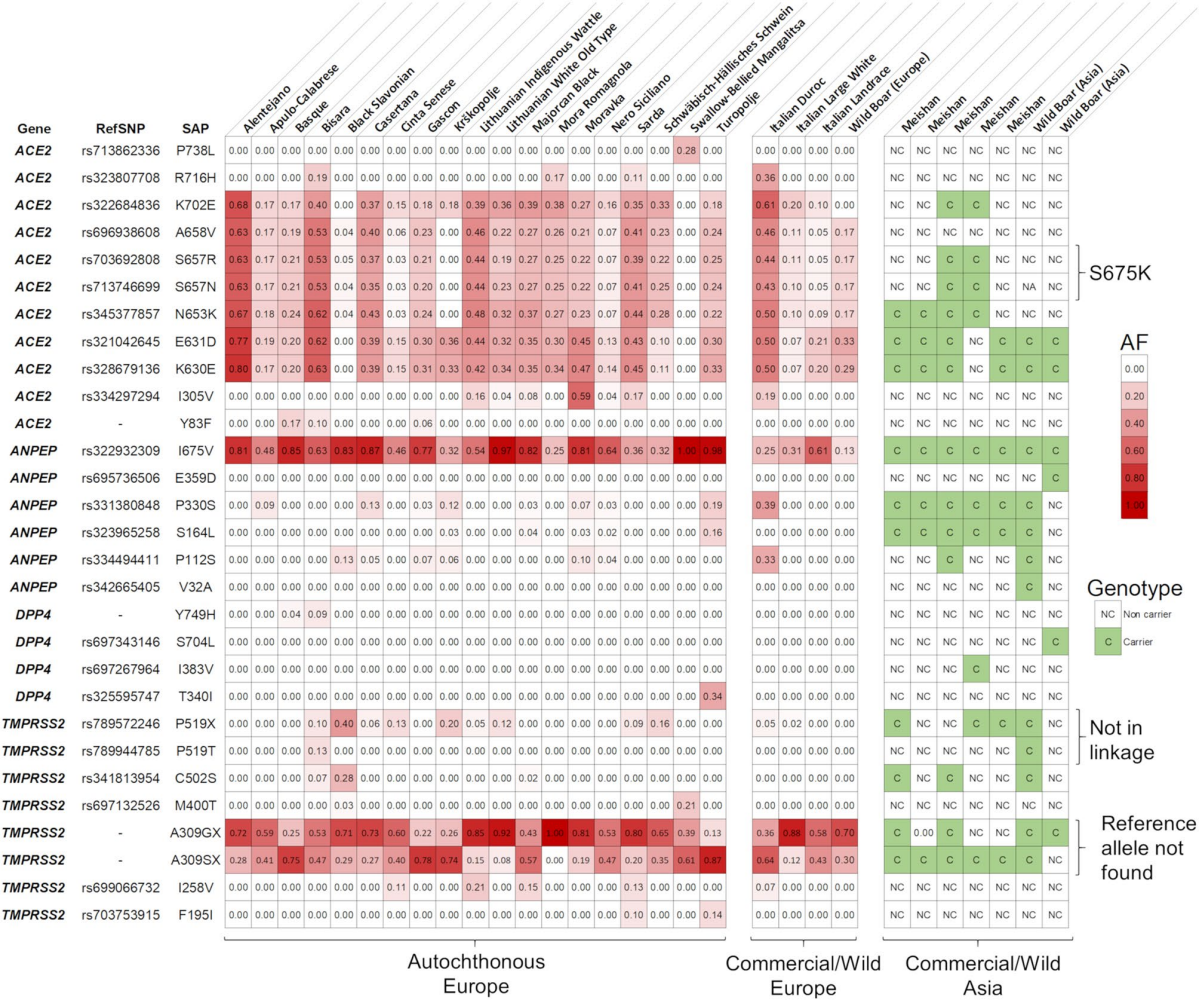
Frequency and genotype information related to the alternative allele of the variants affecting the protein of the four candidate genes (*ACE2*, *ANPEP*, *DPP4* and *TMPRSS2*) in the 23 European and two Asian populations (autochthonous pig breeds, commercial pig breeds and wild boars). Detailed information is provided in Supplementary Table S8. Information for the European breeds and wild boars is obtained from the sequenced DNA pools. Information for the Asian populations is obtained from whole genome sequencing data of individual animals and the right part of the figure reports the carrier status of the alternative allele.

A total of 25 variants affecting the protein sequence of the four candidate genes were identified by mining whole genome resequencing data obtained from the 22 European pig breeds and from the European wild boars (Table 2). Variants were located in all four investigated candidate genes: 11 were in the *ACE2* gene (10 were then considered; see below), four in the *ANPEP* gene, two in the *DPP4* gene and eight in the *TMPRSS2* gene. Allele frequencies of these protein coding variants in the analysed pig breeds and wild boars are reported in Fig. 4 and Supplementary Table S8. All these variants were reported in the European pig breeds and nine segregated in the European wild boars.

**Table 2:** Protein coding variants identified in the European and Asian pig breeds and wild boars.

Gene	SSC^1^	Position^2^	Ref/Alt^3^	AF_Pigs(Europe)_^4^	AF_WB(Europe)_^5^	Meishan^6^	WB(Asia)^7^	RefSNP^8^	SAP^9^	SIFT^10^	SIFT-score^11^
*ACE2*	X	12103359	G/A	0.011	0.000	0/5	0/2	rs713862336	P738L	Deleterious-LC	0.04
*ACE2*	X	12103425	C/T	0.043	0.000	0/5	0/2	rs323807708	R716H	Tolerated-LC	0.08
*ACE2*	X	12105547	T/C	0.276	0.000	2/5	0/2	rs322684836	K702E	Tolerated-LC	1.00
*ACE2*	X	12107234	G/A	0.230	0.167	0/5	0/2	rs696938608	A658V	Tolerated-LC	1.00
*ACE2*	X	12107236	A/T	0.225	0.167	2/5	0/2	rs703692808*	S657K†	Tolerated-LC	0.10
*ACE2*	X	12107237	C/T	0.225	0.167	2/5	0/2	rs713746699*	S657K†	Tolerated-LC	0.09
*ACE2*	X	12107248	A/C	0.254	0.167	4/5	0/2	rs345377857	N653K	Tolerated-LC	1.00
*ACE2*	X	12109953	T/A	0.309	0.333	4/5	2/2	rs321042645	E631D	Tolerated	0.52
*ACE2*	X	12109958	T/C	0.319	0.286	4/5	2/2	rs328679136	K630E	Tolerated	0.40
*ACE2*	X	12120704	T/C	0.061	0.000	0/5	0/2	rs334297294	I305V	Tolerated	0.27
*ACE2*	X	12136848	T/A	0.015	0.000	0/5	0/2	-	Y83F	Tolerated	1.00
*ANPEP*	7	55360022	T/C	0.610	0.133	5/5	2/2	rs322932309	I675V	Tolerated	0.5
*ANPEP*	7	55363723	G/C	0.000	0.000	0/5	1/2	rs695736506	E359D	Deleterious	0.00
*ANPEP*	7	55363906	G/A	0.048	0.000	5/5	1/2	rs331380848	P330S	Tolerated	1.00
*ANPEP*	7	55365462	G/A	0.014	0.000	5/5	1/2	rs323965258	S164L	Tolerated	0.27
*ANPEP*	7	55365619	G/A	0.033	0.000	1/5	1/2	rs334494411	P112S	Tolerated	0.66
*ANPEP*	7	55365858	T/C	0.000	0.000	0/5	1/2	rs342665405	V32A	Tolerated	0.09
*DPP4*	15	68673354	A/G	0.005	0.000	0/5	0/2	–	Y749H	Tolerated	0.70
*DPP4*	15	68676800	C/T	0.000	0.000	0/5	1/2	rs697343146	S704L	Deleterious	0.00
*DPP4*	15	68696930	A/G	0.000	0.000	1/5	0/2	rs697267964	I383V	Deleterious	0.04
*DPP4*	15	68704861	G/A	0.016	0.000	0/5	0/2	rs325595747	T340I	Tolerated	0.16
*TMPRSS2*	13	204877719	A/–	0.297	0.000	3/5	1/2	rs789572246	P519X	–	–
*TMPRSS2*	13	204877721	G/T	0.005	0.000	0/5	1/2	rs789944785	P519T	–	–
*TMPRSS2*	13	204877772	A/T	0.015	0.000	2/5	1/2	rs341813954	C502S	Deleterious-LC	0.04
*TMPRSS2*	13	204878494	A/G	0.013	0.000	0/5	0/2	rs697132526	M400T	Tolerated	0.58
*TMPRSS2*	13	204881920	G/GC	0.598	0.700	2/5	2/2	–	A309GX^§^	–	–
*TMPRSS2*	13	204881920	G/GT	0.402	0.300	5/5	1/2	–	A309SX^§^	–	–
*TMPRSS2*	13	204883347	T/C	0.030	0.000	0/5	0/2	rs699066732	I258V	Deleterious	0.02
*TMPRSS2*	13	204887942	A/T	0.011	0.000	0/5	0/2	rs703753915	F195I	Deleterious	0.02

^1^*Sus scrofa* chromosome; ^2^ Genomic coordinate on the Sscrofa11.1 reference genome; ^3^ Reference/Alternative alleles; ^4^ Frequency of the alternative allele in European pigs (estimated from sequencing data); ^5^ Frequency of the alternative allele in European wild boars (estimated from sequencing data); ^6^ Number of Meishan pigs carrying the variants; ^7^ Number of Asian wild boars carrying the variant; ^8^ dbSNP identification number; ^9^ Single Amino-acid Polymorphism. Protein coordinates refer to UniProtKB accession number listed in Table 1; ^10^ SIFT prediction. LC means low confidence prediction; ^11^ SIFT prediction score.

*Variants rs703692808 and rs713746699, both affecting residue S657, are in complete linkage disequilibrium resulting in the SAP p.S675K†. ^§^ The reference allele was not present in our sequencing. data.

**Figure 5:**
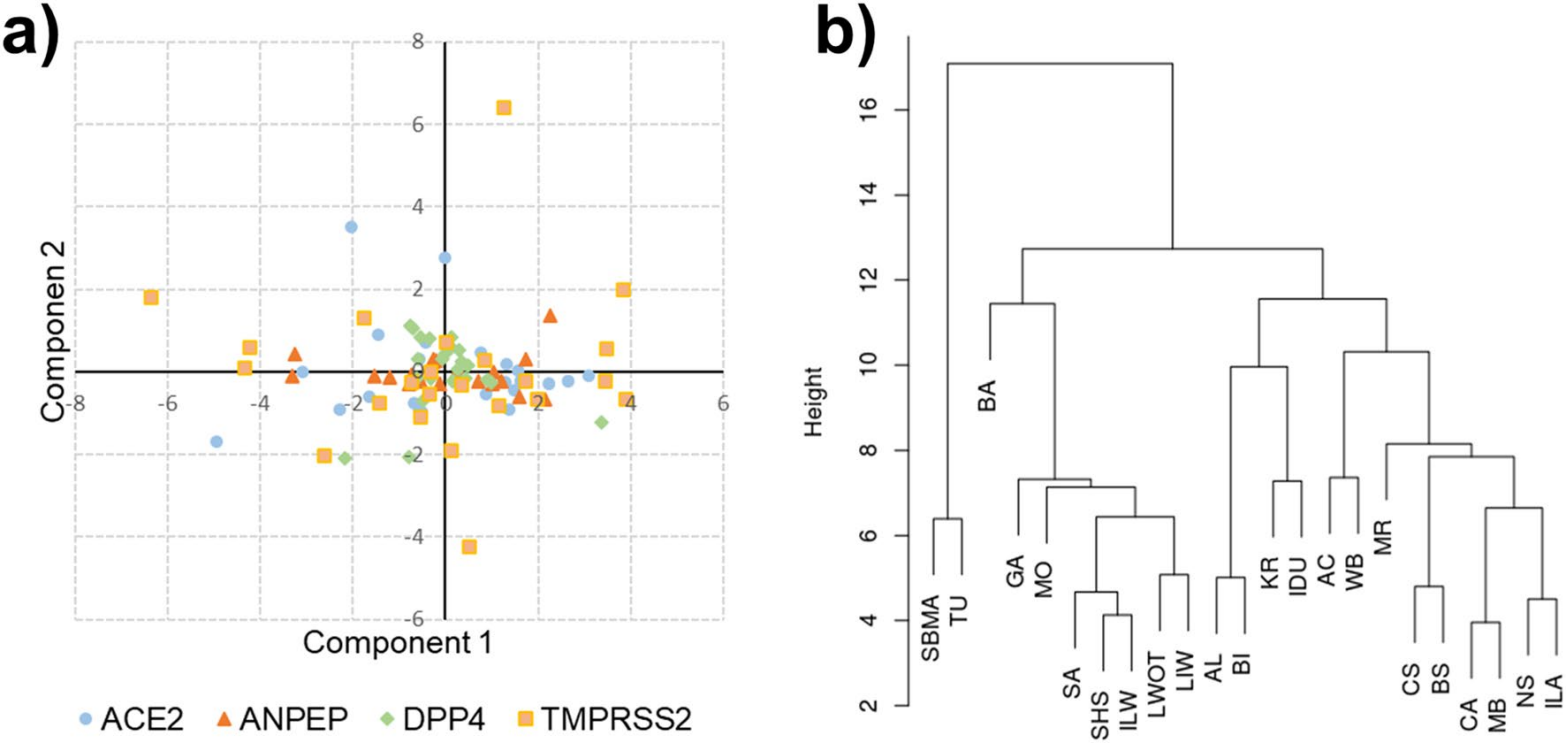
(**a**) Over-imposed multidimensional scaling (MDS) plots and (**b**) cluster analysis of European pig breeds and wild boars determined with information on the polymorphic sites in the *ACE2*, *ANPEP*, *DPP4* and *TMPRSS2* genes. Acronyms of the breed name are given in Fig. 2 and Supplementary Table S1.

Based on this information, European breeds and wild boars were represented in multidimensional scaling plots that showed some contrasting differences among breeds for the information derived by the four genes separately (Fig. 5a). Pig populations were more dissimilar when considering the *TMPRSS2* gene, as points in the plot (i.e. populations) did not form a very compact cloud.

Cluster analysis (Fig. 5b) highlighted similarities among breeds that resembled in part their geographical distribution, including (i) two Lithuanian breeds (Lithuanian indigenous wattle and Lithuanian White old type) and (ii) two Portuguese breeds (Alentejana and Bísara). Wild boars clustered together with Apulo-Calabrese breed. It is worth to note that two breeds from the Balkan Peninsula (Swallow-Bellied Mangalitsa and Turopolje) formed a small cluster completely separated from the rest of the European breeds/populations.

In the porcine *ACE2* gene, as two SNPs (rs703692808 and rs713746699) affect the same residue S657 and that manual inspection of sequenced reads highlighted complete linkage disequilibrium between these two polymorphic sites, they were considered as one variant which caused a novel SAP (p.S675K). Another novel protein coding variant in this gene (X:g.12136848 T > A; p.Y83F) was detected only in Gascon (alternative allele frequency, AF = 0.056), Basque (AF = 0.172) and Bísara (AF = 0.095) breeds.

A novel variant was also identified in the DPP4 protein (15:g.68673354A > G; p.Y749H). The alternative allele was detected only in Basque (AF = 0.04) and Bísara (AF = 0.09) breeds.

A few frameshift mutations were identified in the *TMPRSS2* gene. Variant rs789572246 (13:g. 204877719del) introduces a stop gain codon (p.P519X) near the C-terminal end of the protein, outside the peptidase domain (Fig. 3). The P519 allele was also affected by a second missense variation (rs789944785). A manual inspection of sequenced reads highlighted that these two variants (rs789572246 and rs789944785) were not in complete linkage disequilibrium. Two other novel frameshift mutations (13:g.204881920_20488192insT and 13:g.204881920_20488192insG) would completely change the peptidase coding region of the canonical gene transcript. However, considering an alternative transcript for this gene (transcript ENSSSCT00000026685.3; UniProtKB I3LBF8), these two variants would be annotated as splice donors (as they might change the 2^nd^ base pair region at the 5′-end of an intron). It is worth to mention that at this position, the reference allele was not found in any resequencing dataset in which, instead, all three genotypes insG/insG, insG/insT and insT/insT were called.

Mining whole genome resequencing data retrieved from the Chinese Meishan breed and from Asian wild boars identified other four variants affecting protein sequences (DPP4: p.I383V and p.S704L; ANPEP: p.V32A and p.E359D). Considering also the other variants described above for the European breeds and wild boars, a total of 15 and 14 variants affecting proteins were identified in the Chinese Meishan breed and in the Asian wild boars, respectively (Table 2 and Fig. 4).

#### Putative functional effects of the porcine protein variants

For a comprehensive analysis of the effects of protein coding variants in the four analysed genes, the 29 variants affecting proteins and identified in the European pig breeds and wild boars and in the Asian pig populations (described above) were combined with information on polymorphic sites available in the Ensembl database for the same genes. The Ensembl database reported a total of 60 functional coding variants (10 in the *ACE2* gene, 28 in the *ANPEP* gene, 9 in the *DPP4* gene and 13 in the *TMPRSS2* gene) that combined with the mentioned variants accounted for a total of 64 variants affecting the protein encoded by the four genes (11 in the *ACE2* gene, 28 in the *ANPEP* gene, 10 in the *DPP*4 gene and 15 in the *TMPRSS2* gene; Supplementary Table S9). Figure 3 shows the position of all these variants.

Of the 11 ACE2 protein coding variants, p.P738L was the only one predicted to be deleterious (low confidence). Variants affecting the residues p.N653, p.S657 and p.A658 were located in a protein region interacting with the ADAM17 sheddase whereas variants of the residues p.K702 and p.R716 belong to a domain interacting with the serine proteases TMPRSS1 and TMPRSS2 (Supplementary Table S4). The novel variant p.Y83F detected only in a few European pig breeds (i.e. Gascon, Basque and Bísara) is located within a protein region (human M82-Y83-P84) suggested to participate in SARS-CoV-2 S-protein association^34^.

Of the 27 ANPEP protein coding variants, 22 were classified as tolerated, four were classified as deleterious and one was a frameshift variant (rs431825257) at the C-terminal end of the protein. Based on annotations coming from the human ANPEP protein, none of these SAPs affected sites were relevant for the virus-host interaction or for the functional activity of the protein (Supplementary Table S5). Porcine variants p.M663V, p.F645S, p.A647V and p.R651Q were located in a protein region not homologous to the human protein (i.e. they were included in an alignment gap).

Two out of ten DPP4 protein coding variants were predicted to be deleterious whereas the other seven missense mutations were classified as tolerated. A stop gained variant that eliminates 60 amino acids of the C-terminal end was also identified among the annotated variants in Ensembl. Key sites identified in the comparative analysis did not overlap with any of these variants (Supplementary Table S6). However, the variants p.I383V (p.L316^Human^) and p.A409V (p.A342 ^Human^) were close to the p.R317 ^Human^, p.R336 ^Human^, p.I346 ^Human^ and p.Q344 ^Human^ residues that constitute the MERS-CoV receptor-binding domain (Supplementary Table S7;^84^).

TMPRRS2 protein was affected by a total of 12 missense substitutions (5 tolerated, 6 deleterious and one not classified) and three frameshift mutations. Based on annotations coming from the human TMPRRS2 protein, the variant p.I258V (p.I256^Human^ , Supplementary Table S6) may affect the proteolytic cleavage site (human R255-I256 bond), where auto-cleavage of TMPRSS2 occurs at p.R255 resulting in the release of the active protease^85^.

## Discussion

Genetic resistance to diseases is a complex trait that is re-emerging as a fundamental objective for sustainable programs in animal breeding and selection plans in all livestock species. As a medium to long term selection goal, this objective should be considered as part of a “One Health” strategy that requires more resistant or less susceptible animals to diseases that could be passed to the humans or that could be derived from humans. A few cases, caused by viruses, that also involved the pig in this two-directions transmission route, have been already described (e.g.^86^). Conservation strategies of animal genetic resources should also consider the level of variability within breeds and populations conferring resistance or determining susceptibility to diseases in the context of a global “One Health” perspective.

In animals, genetic resistance to diseases cannot be easily measured and monitored and for these reasons it is difficult to identify any appropriate phenotypic traits as descriptors or proxies of an animal state (related to the diseased or susceptible condition) useful for their inclusion in breeding programs^87^. Alternative strategies or shortcuts that use DNA markers in linkage disequilibrium to causative variants or directly implicated in conferring different levels of susceptibility/resistance or that could be involved (as part of the host response or driven mechanisms) in the infection processes, have been proposed^88^. One of the problems encountered in this strategy is that genetic resistance to diseases is usually a complex quantitative trait that should be considered according to the type of infection agent. Other questions related to this strategy are how it is possible to fill the gaps among the level of the natural genetic variability segregating in the animal populations, the relevance and the effects of these variants in conferring a desired effect against the pathogenic agents and the potential genetic progress against a particular disease that could be achieved (based on the segregating variability). Results that could be obtained in this context can also define risk levels in different populations, as already demonstrated for some diseases in other livestock species (e.g.^89^).

Genomic technologies are opening new opportunities to analyse the host genome at a large scale and then to identify potential candidate mutations conferring resistance to diseases by applying comparative genome analyses across species. This approach takes the advantage from what is known in one species and transfers information in another one. Even if caution should be applied for the interpretation of results, our study provided some information in this direction by describing variability in a few candidate genes of the host (the pig) genome. Whole genome resequencing data that we have generated for many pig genetic resources and the comparative approach that we applied in this study can be further expanded by analysing several other genes for other similar contexts by targeting other diseases and related potential genetic resistance.

In this study, the selected host genes (*ACE2*, *ANPEP*, *DPP4* and *TMPRSS2*) are well known to be involved in the infection mechanisms of coronaviruses: three of them encode for receptors of a few viruses of this group and another one encodes for a key proteinase involved in the initiation of the infection after the invasion of the host susceptible cells^32–43^. The comparative analysis was based on what is known for the human corresponding gene products. The extensive genomic data that we mined in pigs gave the possibility to identify the most frequent variants that can impact on the structure of the encoded proteins.

In many cases of coronavirus infection mechanisms, the entry into the target cell is mediated by the interaction between some cellular receptors and the surface spike (S) glycoprotein^20^. Few of these variants might change the 3D structure or the function of the protein domain in which they are inserted and may potentially modify, at least in part, their role in the infection routes of the targeted coronaviruses in pigs. It is worth to mention that most of the DNA polymorphisms identified in the three genes are located in non-coding regions or do not affect the encoded proteins. It could be possible that some of these variants play regulatory roles but here we did not analyze the sequencing data for this purpose. Gene expression analyses in porcine target tissues would be needed to evaluate the role of these variants in altering the expression of these genes and, in turn, to potentially affect the level of susceptibility to the infection from coronaviruses of pigs with different genotypes.

We studied a large number of autochthonous pig breeds that constitute important genetic resources in Europe. Mutations that we identified in the investigated genes enriched substantially the list of polymorphic sites already described in the *Sus scrofa* for these loci. A large contribution for novel variants derived from the *ACE2* gene. All polymorphisms in the four genes together and their frequencies estimated in 23 European pig populations (22 breeds and one wild boar population) were able to identify substantial differences that made it possible to obtain meaningful clusters of these populations.

Among the 11 variants identified in the ACE2 protein, seven (p.Y83F, p.N653, p.S657, p.A658, p.K702, p.R716 and p.P738L) could potentially modify the protein function. Their effects could be inferred from the information retrieved from the in silico analyses (from SIFT and from their position in specific domains). Particularly, a novel variant (p.Y83F), identified only in a few autochthonous European breeds (Gascon, Basque and Bísara) raised in France and in Portugal, might change the potential association between SARS-CoV-2 S-protein and the host receptor. All studies that thus far have investigated the susceptibility of the pig to SARS-CoV-2 did not consider the possibility of intraspecies variability in the ACE2 receptor protein^12,60,61^ that, actually, exists and could be the source of potential variability in the response to artificial infection experiments. Therefore, in such studies it will be important to report results with a sequence characterization of the host receptor and other key proteins involved in the progression of the infections.

Other potential functional variants were identified in the remaining three proteins. Five of the 27 ANPEP protein variants, two out of 10 DPP4 single amino acid substitutions and nine out of 15 TMPRRS2 protein missense substitutions or frameshift mutations could be deleterious or might change the protein structure and functions. It will be important to evaluate, with in vitro experiments, the role of these variants in the corresponding protein function, including for the receptors, their affinity with the coronavirus S-proteins. These analyses will give the opportunity to also describe the interaction between host variants and with virus variants that could further complicate the infection mechanisms and related pathogenic effects.

The comparative analysis with the human corresponding proteins will be also useful to further acquire elements to describe the pig as a valuable animal model to define genetic mechanisms associated to disease resistance and susceptibility.

Genomic analyses of other breeds and populations could identify additional variants in these four genes that might have a functional relevance, providing a general picture of the variability at these loci. The different levels of variability for these genes can contribute, at least in part, to the potential genetic progress that could be reached against coronavirus infections in pigs once it is established a direct relationship between variants and virus determined diseases. Additional host genes might be also involved in the infection mechanisms of coronaviruses in pigs as gene expression analyses have demonstrated^90^. Moreover, the genetic characterization at the selected loci and additional genes in large number of genetic resources might provide information useful to define how the different breeds could contribute to these aims. Marker assisted selection programs designed to increase genetic resistance to coronaviruses could be based on some of the described polymorphic sites if it will be demonstrated their role in affecting susceptibility of the *Sus scrofa* species. The obtained results will constitute a first step towards the inclusion of conservation and selection programs based on genomic information in this livestock species as part of a comprehensive “One Health” approach against coronaviruses. Risk analysis for coronavirus infections might also consider the variability of the host genome whose level is different across breeds and populations, as it might be derived from their genetic histories.

## Supplementary Information

Supplementary Information.

## Data Availability

Sequence data generated and analysed in the current study from DNA pools are available in the EMBL-EBI European Nucleotide Archive (ENA) repository (http://www.ebi.ac.uk/ena), under the study accession PRJEB36830. From the same repository we retrieved sequence data of five Meishan pigs (samples: ERS804949, ERS804950, ERS804951, ERS804953 and ERS804955) and two Asian wild boars (samples: ERS804971 and ERS805009) deposited with the study accession PRJEB9922. The datasets generated and/or analysed during the current study are available from the corresponding author on reasonable request.
