# Supplementary material for: Describing variability in pig genes involved in coronavirus infections for a One Health perspective in conservation of animal genetic resources
Source: Sci Rep. 2021 Feb 9;11:3359. doi: 10.1038/s41598-021-82956-0 (PMC7873263; doi:10.1038/s41598-021-82956-0)
Supplement: Supplementary file 1 — Supplementary Information. [file 41598_2021_82956_MOESM1_ESM.pdf]

Supplementary material

Describing variability in pig genes involved in coronavirus infections for a One Health perspective in conservation of animal genetic resources

Samuele Bovo, Giuseppina Schiavo, Anisa Ribani, Valerio J. Utzeri, Valeria Taurisano, Mohamad Ballan, Maria Muñoz, Estefania Alves, Jose P. Araujo, Riccardo Bozzi, Rui Charneca, Federica Di Palma, Ivona Djurkin Kušec, Graham Etherington, Ana I. Fernandez, Fabián García, Juan García-Casco, Danijel Karolyi, Maurizio Gallo, José Manuel Martins, Marie-José Mercat, Yolanda Núñez, Raquel Quintanilla, Čedomir Radović, Violeta Razmaite, Juliette Riquet, Radomir Savić, Martin Škrlep, Graziano Usai, Christoph Zimmer, Cristina Ovilo, Luca Fontanesi

Supplementary Table S1. Details on the analyzed animals and investigated breeds.

Breed name	Acronym of the breed name	Native Name	Alternative name	Country / Geographic region	Population	Additional Info
Alentejana	AL	Alentejano, Alentejana	Alentejano	PT / Southwest of the Iberian Peninsula	6464 breeding sows and 510 boars distributed by 137 herds (End of 2017).	https://www.intechopen.com/books/european-local-pig-breeds-diversity-and-performance-a-study-of-project-treasure/alentejano-pig
Apulo-Calabrese	AC	Apulo Calabrese	Apulo Calabrese, Calabrese, Nero Abruzzese, Nero Calabrese, Nero dei Lepini, Nero dei Monti Dauni Meridionali, Nero dei Monti Lepini, Nero di Calabria, Nero di Capitanata, Nero Lucano, Nero Maremmano, Nero Pugliese, Nero Reatino, Pugliese	IT / Central-South of Italy (Lazio, Basilicata and Calabria regions)	489 breeding sows and 93 boars distributed in 45 registered farms (August 2015).	https://www.intechopen.com/books/european-local-pig-breeds-diversity-and-performance-a-study-of-project-treasure/apulo-calabrese-pig
Basque	BA	Basque	Basque<br>Pie Noir du Pays Basque<br>Bigourdan<br>Béarnais<br>Basco-Béarnais<br>Navarrin	FR / Basque Country, a region located in the South-West of France and across Spain border.	580 breeding sows distributed in 28 registered farms (January 2017).	https://www.intechopen.com/books/european-local-pig-breeds-diversity-and-performance-a-study-of-project-treasure/basque-pig
Bísara	BI	Bísaro, Bísara	Bísaro	PT / North of Portugal	5460 breeding sows and 520 boars distributed in189 registered farms (August 2017)	https://www.intechopen.com/books/european-local-pig-breeds-diversity-and-performance-a-study-of-project-treasure/b-saro-pig
Black Slavonian	BS	Black Slavonian	Fajferica	HR / East Croatia	1930 breeding sows and 242 boars distributed in 209 registered farms (December 2017).	https://www.intechopen.com/books/european-local-pig-breeds-diversity-and-performance-a-study-of-project-treasure/black-slavonian-crna-slavonska-pig
Casertana	CA	Casertana	Casertana, Maiale di Teano, Teanese, Pelatella	IT / Central-South of Italy (Campania and Molise regions)	545 breeding sows and 20 boars distributed in 20 registered farms (August 2015).	https://www.intechopen.com/books/european-local-pig-breeds-diversity-and-performance-a-study-of-project-treasure/nero-casertano-pig

Cinta Senese	CS	Cinta Senese	Cinta Senese,<br>Cinta,<br>Cinto, Cinto Toscano,<br>Cinturello Umbro,<br>Cinturino Umbro,<br>Siena Belted	IT / Central Italy (Tuscany region)	5000 animals distributed in 140 farms	https://www.intechopen.com/books/european-local-pig-breeds-diversity-and-performance-a-study-of-project-treasure/cinta-senese-pig
Gascon	GA	Gascon	Gascon	FR / Foot of the Pyrénées mountains in the southwest of France.	1423 breeding sows and 177 breeding males distributed in 64 registered farms (year 2017).	https://www.intechopen.com/books/european-local-pig-breeds-diversity-and-performance-a-study-of-project-treasure/gascon-pig
Krškopolje	KR	Krškopoljski prašič	Krškopolje pig	SI / Slovenia	311 breeding sows and 60 boars distributed in 130 registered farms (August 2015).	https://www.intechopen.com/books/european-local-pig-breeds-diversity-and-performance-a-study-of-project-treasure/kr-kopoljski-pra-i-kr-kopolje-pig-
Lithuanian Indigenous Wattle	LIW	Lietuvos vietines	Lithuanian indigenous wattle	LT / Central part of Lithuania, Baisogala, Radviliškis district	43 breeding sows and 11 boars distributed in 2 registered farms (December 2017)	https://www.intechopen.com/books/european-local-pig-breeds-diversity-and-performance-a-study-of-project-treasure/lietuvos-vietin-lithuanian-indigenous-wattle-pig
Lithuanian White Old Type	LWOT	Senojo tipo Lietuvos baltosios	Old type Lithuanian White	LT / Baisogala, Radviliškis district, in the central part of Lithuania	93 breeding sows and 19 boars distributed in 3 registered farms (December 2017).	https://www.intechopen.com/books/european-local-pig-breeds-diversity-and-performance-a-study-of-project-treasure/lietuvos-baltosios-senojo-tipo-lithuanian-white-pig
Majorcan Black	MB	Negre Mallorquí	Black Majorcan<br>Porc Negre Mallorquí<br>Cerdo Negro Mallorquín	ES / Balearic Islands	969 breeding sows and 54 boars distributed in 59 registered farms (August, 2016).	https://www.intechopen.com/books/european-local-pig-breeds-diversity-and-performance-a-study-of-project-treasure/negre-mallorqu-majorcan-black-pig
Mora Romagnola	MR	Mora Romagnola	Mora Romagnola	IT / North of Italy (Romagna region)	270 breeding sows and 67 boars distributed in the 31 registered farms (August 2015).	https://www.intechopen.com/books/european-local-pig-breeds-diversity-and-performance-a-study-of-project-treasure/mora-romagnola-pig
Moravka	MO	Moravka	Moravka	RS / Morava Valley of central Serbia	265 breeding sows and 15 boars distributed in 25 registered farms (February 2018).	https://www.intechopen.com/books/european-local-pig-breeds-diversity-and-performance-a-study-of-project-treasure/moravka-pig
Nero Siciliano	NS	Nero Siciliano	Nero dei Nebrodi,<br>Nero delle Madonie,<br>Nero dell'Etna	IT / Sicily island	1103 breeding sows and 124 boars distributed in 87 registered farms (August 2015).	https://www.intechopen.com/books/european-local-pig-breeds-diversity-and-performance-a-study-of-project-treasure/nero-siciliano-pig
Sarda	SA	Sarda	Sarda	IT / Sardinia island	61 breeding sows and 20 boars distributed in 13 registered farms.	https://www.intechopen.com/books/european-local-pig-breeds-diversity-and-performance-a-study-of-project-treasure/sarda-pig
Schwäbisch-Hällisches Schwein	SHS	Schwäbisch-Hällisches Schwein	Schwaebisch-Haellisches Schwein<br>Swabian Hall pig,<br>Swabian Hall swine	DE / Area of Schwäbisch Hall	350 breeding sows and 35 boars distributed in 15 registered farms (August 2015).	https://www.intechopen.com/books/european-local-pig-breeds-diversity-and-performance-a-study-of-project-treasure/schw-bisch-h-llisches-pig

Swallow-Bellied Mangalitsa	SBMA	Mangulica	Mangalitsa	RS / Serbia	925 sows, 605 gilts and 42 boars distributed in 67 registered farm (End of 2017)	https://www.intechopen.com/books/european-local-pig-breeds-diversity-and-performance-a-study-of-project-treasure/mangalitsa-swallow-belly-mangalitsa-pig
Turopolje	TU	Turopoljska svinja	Turopolje pig	HR / West Croatia	116 breeding sows and 14 boars distributed in 12 registered farms (August 2016).	https://www.intechopen.com/books/european-local-pig-breeds-diversity-and-performance-a-study-of-project-treasure/turopolje-pig-turopoljska-svinja-
Italian Duroc	IDU	Duroc Italiana	Duroc Italiana	IT	-	www.anas.it
Italian Large White	ILW	Large White Italiana	Large White Italiana	IT	-	www.anas.it
Italian Landrace	ILA	Landrace Italiana	Landrace Italiana	IT	-	www.anas.it
Wild Boar	WB	Wild Boar	Wild Boar	IT	-	-

Supplementary Table S2. Summary statistics of whole-genome resequencing data.

Breed	Country	No. of read pairs	No. of animals	Breadth of coverage (%)	Depth of coverage (×)
**Autochthonous (Europe)**					
Alentejana	PT	419,690,476	35	98.42	41.98
Apulo-Calabrese	IT	418,529,727	35	98.49	42.12
Basque	FR	407,698,128	30	98.35	39.55
Bísara	PT	415,284,437	35	98.51	42.44
Black Slavonian	HR	405,316,112	35	98.51	40.61
Casertana	IT	435,598,516	35	98.50	43.61
Cinta Senese	IT	422,120,850	35	98.47	42.42
Gascon	FR	408,764,207	30	98.47	41.10
Krškopolje	SI	404,204,144	35	98.52	40.80
Lithuanian Indigenous Wattle	LT	409,935,460	35	98.48	41.99
Lithuanian White Old Type	LT	405,822,217	35	98.45	41.62
Majorcan Black	ES	414,314,159	35	98.48	41.92
Mora Romagnola	IT	411,095,541	35	98.45	41.21
Moravka	RS	413,100,992	35	98.49	42.27
Nero Siciliano	IT	405,812,223	35	98.44	38.92
Sarda	IT	442,035,147	35	98.51	44.32
Schwäbisch-Hällisches Schwein	DE	428,982,876	35	98.48	42.69
Swallow-Bellied Mangalitsa	RS	416,663,891	35	98.45	41.08
Turopolje	HR	416,663,891	35	98.36	42.61
**Commercial (Europe)**					
Italian Duroc	IT	420,384,723	35	98.51	41.91
Italian Large White	IT	450,673,024	35	98.38	45.24
Italian Landrace	IT	442,780,637	35	98.48	44.35
**Wild Boar (Europe)**	IT	164,203,815	35	98.20	11.74
Meishan (Asia)	Asia	153,384,747	1	97.86	12.16
Meishan (Asia)	Asia	159,324,510	1	97.95	12.54
Meishan (Asia)	Asia	154,524,714	1	97.20	11.92
Meishan (Asia)	Asia	118,665,143	1	97.65	9.35
Meishan (Asia)	Asia	161,517,220	1	97.92	12.72
**Wild Boar (Asia)**	Asia	196,283,001	1	98.12	15.02
**Wild Boar (Asia)**	Asia	84,751,336	1	92.31	6.75

Supplementary Table S3. Number of variants in candidate genes discovered in the analysis of European pig breeds.

	Variant type			Variant location[§]						Variant consequence			
**Gene***	**SNPs**	**INDELs**		**Flanking (3'-UTR)**	**3'-UTR**	**Exon**	**Intron**	**5'-UTR**	**Flanking (5'-UTR)**	**Splice region**	**Synonymous**	**Missense**	**Frameshift**
ACE2													
	750	87	Novel	14	0	2	374	0	71	2	1	1	0
			dbSNP	33	2	21	428	0	0	3	11	10	0
			**Total**	47	2	23	802	0	71	5	12	11	0
ANPEP													
	161	12	Novel	6	0	0	11	0	3	0	0	0	0
			dbSNP	25	4	7	98	1	35	1	3	4	0
			**Total**	31	4	7	109	1	38	1	3	4	0
DPP4													
	401	59	Novel	5	0	3	53	1	3	0	2	1	0
			dbSNP	21	5	3	344	4	18	1	2	1	0
			**Total**	26	5	6	397	5	21	1	4	2	0
TMPRSS2													
	687	72	Novel	7	3	0	54	0	6	0	0	0	1
			dbSNP	91	19	18	474	0	87	3	11	5	0
			**Total**	98	22	18	526	0	93	3	11	5	1

* The identifier of the canonical transcript and gene coordinates are reported in Table 1. Data consider also additional flanking regions of 5 kbp.

§ The total sum is not equal to the no. of called variants (variant type) as they can co-locate or have multiple consequences as predicted with VEP.

Supplementary Table S4. ACE2 residues critical for protein function and coronaviruses pathogenesis.

Interaction sites[1]

HSA*	S19	Q24	A25	K26	T27	F28	D30	K31	H34	E35	E37	D38	Y41	Q42	L45	K68	L79	M82	Y83	N90	Q325	E329	N330	N339	K353	G354	D355	R357	M383	P389	R393	S425	P426	D427	R559
SSC^	.	L24	.	.	F27	.	E30	.	L34	.	.	.	.	.	.	.	I79	T82	.	T90	.	N329	.	.	.	.	.	.	.	.	.	P425	.	.	S559
NGS§	.	.	.	.	.	.	.	.	.	.	.	.	.	.	.	.	.	.	F83	.	.	.	.	.	.	.	.	.	.	.	.	.	.	.	.

Binding/Active sites[2]

	Binding site	Binding site	Binding site	Binding site	Binding site	Metal binding	Active site	Metal binding	Metal binding	Binding site	Binding site	Binding site	Binding site
HSA	R169	R273	H345	P346	T371	H374	E375	H378	E402	W477	K481	H505	Y515
SSC	.	.	.	.	.	.	.	.	.	.	.	.	.
NGS	.	.	.	.	.	.	.	.	.	.	.	.	.

Cleavage by ADAM17[2]

HSA	R652	Q653	Y654	655F	L656	K657	V658	K659
SSC	.	N653	.	.	S656	S657	A658	.
NGS	.	.	.	.	.	N657 R657	.	.

Cleavage by TMPRSS1 and TMPRSS2[2]

HSA	R697	T698	E699	V700	E701	K702	A703	I704	R705	M706	S707	R708	S709	R710	I711	N712	D713	A714	F715	R716
SSC	.	S698	D699	.	.	.	.	.	S705	.	.	.	.	.	.	.	.	.	.	.
NGS	.	.	.	.	.	E702	.	.	.	.	.	.	.	.	.	.	.	.	.	H716

Glycosylation sites[3]

HSA	N53	N90	N103	N322	N432	N546
SSC	.	T90	S103	.	.	.
NGS	.	.	.	.	.	.

* Residues are based on the human UniProtKB entry Q9BYF1.
^ Residues are based on the porcine UniProtKB entry K7GLM4. A dot represents an identical residue where residues differing between human and pig are reported.
§ Variants identified in the next generation sequencing datasets. Residues are reported. A dot represents an identical residue.
[1] UniProtKB annotations, Lan et al. (2020), Shang et al. (2020), Benetti et al. (2020), Sun et al. (2020), Damas et al. (2020), Li W et al. (2005), Li F et al (2005), Luan et al. (2020), Cao et al. (2020)
[2] UniProtKB annotations, Towler et al. (2004)
[3] UniProtKB annotations, Towler et al. (2004), Sun et al. (2020), Kristiansen et al. (2004), Chen et al. (2009).

Supplementary Table S5. ANPEP residues critical for protein function and coronaviruses pathogenesis. Residues differing between human and pig are underlined.

**Interaction sites[1]**										
HSA*	D288	Y289	V290	E291	K292	Q293	A294	S295	D315	L318
SSC^	Q283	S284	V285	N286	E287	T288	A289	Q290	M310	L313
NGS§	.	.	.	.	.	.	.	.	.	.
**Binding/Active sites[2]**										
	Metal binding	Active site	Metal binding	Metal binding	Site					
HSA	H388	E389	H392	E411	Y477					
SSC	H383	E384	H387	E406	Y472					
NGS	.	.	.	.	.					
**Glycosylation sites[3]**										
HSA	N128	N234	N265	N319	N527	N573	N625	N681	N818	
SSC	N124	N229	S260	N314	D521	N569	N622	N676	Q869	
NGS	.	.	.	.	.	.	.	.	.	

* Residues are based on the human UniProtKB entry P15144.
^ Residues are based on the porcine UniProtKB entry A0A5G2QI26. Residues differing between human and pig are underlined.
§ Variants identified in the next generation sequencing datasets. Residues are reported. A dot represents an identical residue.

1 UniProtKB annotations, Li Z et al. (2019), Wentworth et al. (2019).
2 UniProtKB annotations, Wong A et al. (2012), Kolb et al. (1996).
3 UniProtKB annotations, Wentworth et al. (2019), Wong et al. (2012).

Supplementary Table S6. DPP4 residues critical for protein function and coronaviruses pathogenesis. Residues differing between human and pig are underlined.

**Interaction sites[1]**															
HSA*	L267	P269	Q286	T288	A289	A291	L294	I295	H298	A317	T322	R336	Q344	I346	L392
SSC^	K334	F336	Q353	V355	P356	A358	L361	I362	H365	R384	Y389	R403	Q411	I413	S459
NGS[§]	.	.	.	.	.	.	.	.	.	.	.	.	.	.	.
**Active sites[2]**															
HSA	E205	E206	Y547	S630	D708	H740									
SSC	E272	E273	Y614	S697	D775	H807									
NGS	.	.	.	.	.	.									
**Glycosylation sites[3]**															
HSA	N85	N92	N150	N219	N229	N281	N321	N520	N685						
SSC	N152	N159	N217	N286	N296	S348	N388	H587	N752						
NGS	.	.	.	.	.	.	.	.	.						

*Residues are based on the human UniProtKB entry P27487.
^ Residues are based on the porcine UniProtKB entry A0A5G2Q7G7. Residues differing between human and pig are underlined.
§ Variants identified in the next generation sequencing datasets. Residues are reported. A dot represents an identical residue.
[1] UniProtKB annotations, Wang N et al. (2013), de Wit et al. (2017).
[2] UniProtKB annotations, Kirby et al. (2010).
[3] UniProtKB annotations, Hiramatsu et al. (2003), Rasmussen et al. (2003), Thoma et al. (2003), Meng et al. (2010), Chen R et al. (2009).

Supplementary Table S7. TMPRSS2 residues critical for protein function and coronaviruses pathogenesis.

**Cleavage/Binding/Active sites**[1]								
	Cleavage	Cleavage	Active site	Active site	substrate binding	Active site	substrate binding	substrate binding
HSA*	R255	I256	H296	I346	D435	S441	S460	G462
SSC^	R257	I258	H298	I345	D434	S440	S459	G461
NGS[§]	.	V258		.	.	.	.	.

[*] Residues are based on the human UniProtKB entry O15393.
[^] Residues are based on the porcine UniProtKB entry A0A287AFA0. Residues differing between human and pig are underlined.
[§] Variants identified in the next generation sequencing datasets. Residues are reported. A dot represents an identical residue.

[1] Hussain et al. (2020), Afar et al. (2001), Benetti et al. (2020).

Supplementary Table S8. Allele frequencies of the protein coding variants (alternative allele) in the analysed pig breeds and populations.

						European breeds/populations*																							Asian breeds/populations§						
**Gene**	**SSC**	**Position**	**Ref/Alt**	**RefSNP**	**SAP**	**AL**	**AC**	**BA**	**BI**	**BS**	**CA**	**CS**	**GA**	**KR**	**LIW**	**LWOT**	**MB**	**MR**	**MO**	**NS**	**SA**	**SHS**	**SWMA**	**TU**	**IDU**	**ILW**	**ILA**	**WB**	**ME**	**ME**	**ME**	**ME**	**ME**	**WB**	**WB**
ACE2	X	12103359	G/A	rs713862336	P738L	0.00	0.00	0.00	0.00	0.00	0.00	0.00	0.00	0.00	0.00	0.00	0.00	0.00	0.00	0.00	0.00	0.00	0.28	0.00	0.00	0.00	0.00	0.00	NC	NC	NC	NC	NC	NC	NC
ACE2	X	12103425	C/T	rs323807708	R716H	0.00	0.00	0.00	0.19	0.00	0.00	0.00	0.00	0.00	0.00	0.00	0.00	0.17	0.00	0.00	0.11	0.00	0.00	0.00	0.36	0.00	0.00	0.00	NC	NC	NC	NC	NC	NC	NC
ACE2	X	12105547	T/C	rs322684836	K702E	0.68	0.17	0.17	0.40	0.00	0.37	0.15	0.18	0.18	0.39	0.36	0.39	0.38	0.27	0.16	0.35	0.33	0.00	0.18	0.61	0.20	0.10	0.00	NC	NC	C	C	NC	NC	NC
ACE2	X	12107234	G/A	rs696938608	A658V	0.63	0.17	0.19	0.53	0.04	0.40	0.06	0.23	0.00	0.46	0.22	0.27	0.26	0.21	0.07	0.41	0.23	0.00	0.24	0.46	0.11	0.05	0.17	NC	NC	NC	NC	NC	NC	NC
ACE2	X	12107236	A/T	rs703692808	S657R	0.63	0.17	0.21	0.53	0.05	0.37	0.03	0.21	0.00	0.44	0.19	0.27	0.25	0.22	0.07	0.39	0.22	0.00	0.25	0.44	0.11	0.05	0.17	NC	NC	C	C	NC	NC	NC
ACE2	X	12107237	C/T	rs713746699	S657N	0.63	0.17	0.21	0.53	0.04	0.35	0.03	0.20	0.00	0.44	0.23	0.27	0.25	0.22	0.07	0.41	0.25	0.00	0.24	0.43	0.10	0.05	0.17	NC	NC	C	C	NC	NA	NC
ACE2	X	12107248	A/C	rs345377857	N653K	0.67	0.18	0.24	0.62	0.04	0.43	0.03	0.24	0.00	0.48	0.32	0.37	0.27	0.23	0.07	0.44	0.28	0.00	0.22	0.50	0.10	0.09	0.17	C	C	C	C	NC	NC	NC
ACE2	X	12109953	T/A	rs321042645	E631D	0.77	0.19	0.20	0.62	0.00	0.39	0.15	0.30	0.36	0.44	0.32	0.35	0.30	0.45	0.13	0.43	0.10	0.00	0.30	0.50	0.07	0.21	0.33	C	C	C	NC	C	C	C
ACE2	X	12109958	T/C	rs328679136	K630E	0.80	0.17	0.20	0.63	0.00	0.39	0.15	0.31	0.33	0.42	0.34	0.35	0.34	0.47	0.14	0.45	0.11	0.00	0.33	0.50	0.07	0.20	0.29	C	C	C	NC	C	C	C
ACE2	X	12120704	T/C	rs334297294	I305V	0.00	0.00	0.00	0.00	0.00	0.00	0.00	0.00	0.00	0.16	0.04	0.08	0.00	0.59	0.04	0.17	0.00	0.00	0.00	0.19	0.00	0.00	0.00	NC	NC	NC	NC	NC	NC	NC
ACE2	X	12136848	T/A	-	Y83F	0.00	0.00	0.17	0.10	0.00	0.00	0.00	0.06	0.00	0.00	0.00	0.00	0.00	0.00	0.00	0.00	0.00	0.00	0.00	0.00	0.00	0.00	0.00	NC	NC	NC	NC	NC	NC	NC
ANPEP	7	55360022	T/C	rs322932309	I675V	0.81	0.48	0.85	0.63	0.83	0.87	0.46	0.77	0.32	0.54	0.97	0.82	0.25	0.81	0.64	0.36	0.32	1.00	0.98	0.25	0.31	0.61	0.13	C	C	C	C	C	C	C
ANPEP	7	55363723	G/C	rs695736506	E359D	0.00	0.00	0.00	0.00	0.00	0.00	0.00	0.00	0.00	0.00	0.00	0.00	0.00	0.00	0.00	0.00	0.00	0.00	0.00	0.00	0.00	0.00	0.00	NC	NC	NC	NC	NC	NC	C
ANPEP	7	55363906	G/A	rs331380848	P330S	0.00	0.09	0.00	0.00	0.00	0.13	0.00	0.03	0.12	0.00	0.00	0.03	0.00	0.07	0.03	0.00	0.00	0.00	0.19	0.39	0.00	0.00	0.00	C	C	C	C	C	C	NC
ANPEP	7	55365462	G/A	rs323965258	S164L	0.00	0.00	0.00	0.00	0.00	0.00	0.00	0.00	0.03	0.00	0.00	0.04	0.00	0.03	0.02	0.00	0.00	0.00	0.16	0.00	0.00	0.00	0.00	C	C	C	C	C	C	NC
ANPEP	7	55365619	G/A	rs334494411	P112S	0.00	0.00	0.00	0.00	0.13	0.05	0.00	0.07	0.06	0.00	0.00	0.00	0.00	0.10	0.04	0.00	0.00	0.00	0.00	0.33	0.00	0.00	0.00	NC	NC	C	NC	NC	C	NC
ANPEP	7	55365858	T/C	rs342665405	V32A	0.00	0.00	0.00	0.00	0.00	0.00	0.00	0.00	0.00	0.00	0.00	0.00	0.00	0.00	0.00	0.00	0.00	0.00	0.00	0.00	0.00	0.00	0.00	NC	NC	NC	NC	NC	C	NC
DPP4	15	68673354	A/G	-	Y749H	0.00	0.00	0.04	0.09	0.00	0.00	0.00	0.00	0.00	0.00	0.00	0.00	0.00	0.00	0.00	0.00	0.00	0.00	0.00	0.00	0.00	0.00	0.00	NC	NC	NC	NC	NC	NC	NC
DPP4	15	68676800	C/T	rs697343146	S704L	0.00	0.00	0.00	0.00	0.00	0.00	0.00	0.00	0.00	0.00	0.00	0.00	0.00	0.00	0.00	0.00	0.00	0.00	0.00	0.00	0.00	0.00	0.00	NC	NC	NC	NC	NC	NC	C
DPP4	15	68696930	A/G	rs697267964	I383V	0.00	0.00	0.00	0.00	0.00	0.00	0.00	0.00	0.00	0.00	0.00	0.00	0.00	0.00	0.00	0.00	0.00	0.00	0.00	0.00	0.00	0.00	0.00	NC	NC	NC	C	NC	NC	NC
DPP4	15	68704861	G/A	rs325595747	T340I	0.00	0.00	0.00	0.00	0.00	0.00	0.00	0.00	0.00	0.00	0.00	0.00	0.00	0.00	0.00	0.00	0.00	0.00	0.34	0.00	0.00	0.00	0.00	NC	NC	NC	NC	NC	NC	NC
TMPRSS2	13	204877719	GA/G	rs789572246	P519X	0.00	0.00	0.00	0.10	0.40	0.06	0.13	0.00	0.20	0.05	0.12	0.00	0.00	0.00	0.00	0.09	0.16	0.00	0.00	0.05	0.02	0.00	0.00	C	NC	NC	C	C	C	NC
TMPRSS2	13	204877721	G/T	rs789944785	P519T	0.00	0.00	0.00	0.13	0.00	0.00	0.00	0.00	0.00	0.00	0.00	0.00	0.00	0.00	0.00	0.00	0.00	0.00	0.00	0.00	0.00	0.00	0.00	NC	NC	NC	NC	NC	C	NC
TMPRSS2	13	204877772	A/T	rs341813954	C502S	0.00	0.00	0.00	0.07	0.28	0.00	0.00	0.00	0.00	0.00	0.00	0.02	0.00	0.00	0.00	0.00	0.00	0.00	0.00	0.00	0.00	0.00	0.00	C	NC	C	NC	NC	C	NC
TMPRSS2	13	204878494	A/G	rs697132526	M400T	0.00	0.00	0.00	0.00	0.03	0.00	0.00	0.00	0.00	0.00	0.00	0.00	0.00	0.00	0.00	0.00	0.00	0.21	0.00	0.00	0.00	0.00	0.00	NC	NC	NC	NC	NC	NC	NC
TMPRSS2	13	204881920	G/GC	-	A309GX	0.72	0.59	0.25	0.53	0.71	0.73	0.60	0.22	0.26	0.85	0.92	0.43	1.00	0.81	0.53	0.80	0.65	0.39	0.13	0.36	0.88	0.58	0.70	C	0.00	C	NC	NC	C	C

TMPRSS2	13	204881920	G/GT	-	A309SX	0.28	0.41	0.75	0.47	0.29	0.27	0.40	0.78	0.74	0.15	0.08	0.57	0.00	0.19	0.47	0.20	0.35	0.61	0.87	0.64	0.12	0.43	0.30	C	C	C	C	C	C	NC
TMPRSS2	13	204883347	T/C	rs699066732	I258V	0.00	0.00	0.00	0.00	0.00	0.00	0.11	0.00	0.00	0.21	0.00	0.15	0.00	0.00	0.00	0.13	0.00	0.00	0.00	0.07	0.00	0.00	0.00	NC	NC	NC	NC	NC	NC	NC
TMPRSS2	13	204887942	A/T	rs703753915	F195I	0.00	0.00	0.00	0.00	0.00	0.00	0.00	0.00	0.00	0.00	0.00	0.00	0.00	0.00	0.00	0.10	0.00	0.00	0.14	0.00	0.00	0.00	0.00	NC	NC	NC	NC	NC	NC	NC

* Acronyms of the breed name are given in Supplementary Table S1.

§ *ME* indicates Meishan pigs. *C* and *NC* indicate carries and non-carries of the variant.

Supplementary Table S9. All functional coding variants in the porcine *ACE2*, *ANPEP*, *DPP4* and *TMPRSS2* genes detected by mining whole genome resequencing datasets and retrieved from Ensembl, release 100.

Location[1]	Alleles	Variant ID	Gene	Transcript	Consequence	Residue	SIFT	SIFT score	NGS
X:12103359	G/A	rs713862336	*ACE2*	ENSSSCT00000034032.2	missense variant	P738L	deleterious - low confidence	0.04	YES
X:12103425	C/T	rs323807708	*ACE2*	ENSSSCT00000034032.2	missense variant	R716H	tolerated - low confidence	0.08	YES
X:12105547	T/C	rs322684836	*ACE2*	ENSSSCT00000034032.2	missense variant	K702E	tolerated - low confidence	1.00	YES
X:12107234	G/A	rs696938608	*ACE2*	ENSSSCT00000034032.2	missense variant	A658V	tolerated - low confidence	1.00	YES
X:12107236	A/T	rs703692808	*ACE2*	ENSSSCT00000034032.2	missense variant	S657R	tolerated - low confidence	0.10	YES
X:12107237	C/T	rs713746699	*ACE2*	ENSSSCT00000034032.2	missense variant	S657N	tolerated - low confidence	0.09	YES
X:12107248	A/C	rs345377857	*ACE2*	ENSSSCT00000034032.2	missense variant	N653K	tolerated - low confidence	1.00	YES
X:12109953	T/A	rs321042645	*ACE2*	ENSSSCT00000034032.2	missense variant	E631D	tolerated	0.52	YES
X:12109958	T/C	rs328679136	*ACE2*	ENSSSCT00000034032.2	missense variant	K630E	tolerated	0.40	YES
X:12120704	T/C	rs334297294	*ACE2*	ENSSSCT00000034032.2	missense variant	I305V	tolerated	0.27	YES
X:12136848	T/A	-	*ACE2*	-	missense variant	Y83F	tolerated	1.00	YES
7:55351535	C/G	rs321552301	*ANPEP*	ENSSSCT00000086218.1	missense variant	E1015Q	tolerated	0.21	NO
7:55351584	G/T	rs702354052	*ANPEP*	ENSSSCT00000086218.1	missense variant	N998K	deleterious	0.01	NO
7:55351589	C/T	rs80819730	*ANPEP*	ENSSSCT00000086218.1	missense variant	A997T	tolerated	0.17	NO
7:55352761	A/G	rs80840529	*ANPEP*	ENSSSCT00000086218.1	missense variant	L904P	tolerated	0.20	NO
7:55352771	T/G	rs690295979	*ANPEP*	ENSSSCT00000086218.1	missense variant	N901H	deleterious	0.01	NO
7:55359943	A/G	rs694785744	*ANPEP*	ENSSSCT00000086218.1	missense variant	L701P	tolerated	0.31	NO
7:55359949	G/A	rs704508162	*ANPEP*	ENSSSCT00000086218.1	missense variant	T699I	tolerated	0.07	NO
7:55359973	C/T	rs708356135	*ANPEP*	ENSSSCT00000086218.1	missense variant	R691K	tolerated	0.50	NO
7:55359983	C/T	rs694071064	*ANPEP*	ENSSSCT00000086218.1	missense variant	D688N	tolerated	0.46	NO
7:55359986	C/T	rs790254658	*ANPEP*	ENSSSCT00000086218.1	missense variant	E687K	tolerated	0.83	NO
7:55360022	T/C	rs322932309	*ANPEP*	ENSSSCT00000086218.1	missense variant	I675V	tolerated	0.50	YES
7:55360093	C/T	rs332460794	*ANPEP*	ENSSSCT00000086218.1	missense variant	R651Q	tolerated	0.47	YES
7:55360105	G/A	rs696362099	*ANPEP*	ENSSSCT00000086218.1	missense variant	A647V	tolerated	0.29	NO
7:55360111	A/G	rs703234834	*ANPEP*	ENSSSCT00000086218.1	missense variant	F645S	tolerated	0.07	NO
7:55360118	T/C	rs712865931	*ANPEP*	ENSSSCT00000086218.1	missense variant	M643V	tolerated	0.51	NO
7:55360246	T/C	rs705719244	*ANPEP*	ENSSSCT00000086218.1	missense variant	D600G	tolerated	0.38	NO
7:55360249	C/T	rs691486822	*ANPEP*	ENSSSCT00000086218.1	missense variant	R599Q	tolerated	0.59	NO
7:55360253	G/T	rs698244278	*ANPEP*	ENSSSCT00000086218.1	missense variant	L598M	tolerated	0.23	NO
7:55360585	C/T	rs698986521	*ANPEP*	ENSSSCT00000086218.1	missense variant	E567K	tolerated	0.20	NO
7:55363723	C/G	rs695736506	*ANPEP*	ENSSSCT00000086218.1	missense variant	E359D	deleterious	-	YES
7:55363906	G/A	rs331380848	*ANPEP*	ENSSSCT00000086218.1	missense variant	P330S	tolerated	1.00	YES
7:55364439	A/G	rs708466949	*ANPEP*	ENSSSCT00000086218.1	missense variant	V293A	deleterious	0.01	NO

7:55365462	G/A	rs323965258	*ANPEP*	ENSSSCT00000086218.1	missense variant	S164L	tolerated	0.27	YES
7:55365547	C/T	rs711934067	*ANPEP*	ENSSSCT00000086218.1	missense variant	G136S	tolerated	0.27	NO
7:55365619	G/A	rs334494411	*ANPEP*	ENSSSCT00000086218.1	missense variant	P112S	tolerated	0.66	YES
7:55365631	G/T	rs431825257	*ANPEP*	ENSSSCT00000086218.1	frameshift variant	FI107FX	-	-	NO
7:55365631	G/T	rs431825257	*ANPEP*	ENSSSCT00000086218.1	frameshift variant	FI107LX	-	-	NO
7:55365858	A/G	rs342665405	*ANPEP*	ENSSSCT00000086218.1	missense variant	V32A	tolerated	0.09	NO
15:68663912	T/C	rs706766380	*DPP4*	ENSSSCT00000067722.1	missense variant	H771R	deleterious	-	NO
15:68663923	G/T	rs692548435	*DPP4*	ENSSSCT00000067722.1	stop gained	Y767*	-	--	NO
15:68673354	A/G	-	*DPP4*	-	missense variant	Y749H	tolerated	0.70	YES
15:68676800	G/A	rs697343146	*DPP4*	ENSSSCT00000067722.1	missense variant	S704L	deleterious	-	YES
15:68680521	T/C	rs81213853	*DPP4*	ENSSSCT00000067722.1	missense variant	S636G	deleterious	-	NO
15:68694798	G/A	rs704514617	*DPP4*	ENSSSCT00000067722.1	missense variant splice region variant	A409V	tolerated	0.32	NO
15:68696930	T/C	rs697267964	*DPP4*	ENSSSCT00000067722.1	missense variant	I383V	deleterious	0.04	YES
15:68704861	G/A	rs325595747	*DPP4*	ENSSSCT00000067722.1	missense variant	T340I	tolerated	0.16	YES
15:68706434	G/T	rs712862703	*DPP4*	ENSSSCT00000067722.1	missense variant	P301T	deleterious	0.01	NO
15:68710385	A/T	rs706520405	*DPP4*	ENSSSCT00000067722.1	missense variant	N237K	deleterious	0.01	NO
13:204877719	A/-	rs789572246	*TMPRSS2*	ENSSSCT00000041631.2	frameshift variant	P519X	-	-	YES
13:204877721	G/T	rs789944785	*TMPRSS2*	ENSSSCT00000041631.2	missense variant	P519T	-	-	YES
13:204877772	A/T	rs341813954	*TMPRSS2*	ENSSSCT00000041631.2	missense variant	C502S	deleterious - low confidence	0.04	YES
13:204877780	A/G	rs702135491	*TMPRSS2*	ENSSSCT00000041631.2	missense variant	L499P	deleterious - low confidence	0.03	NO
13:204878494	A/G	rs697132526	*TMPRSS2*	ENSSSCT00000041631.2	missense variant	M400T	tolerated	0.58	
13:204881040	A/G	rs690909053	*TMPRSS2*	ENSSSCT00000041631.2	missense variant	W379R	deleterious	-	NO
13:204881905	A/C	rs704726313	*TMPRSS2*	ENSSSCT00000041631.2	missense variant	L314W	deleterious	-	NO
13:204881920	G/GC	-	*TMPRSS2*	-	frameshift variant splice region variant	A309G/X		-	YES
13:204881920	G/GT	-	*TMPRSS2*	-	frameshift variant splice region variant	A309G/X		-	YES
13:204881921	C/T	rs337297302	*TMPRSS2*	ENSSSCT00000041631.2	missense variant	A309T	tolerated	0.06	NO
13:204883347	T/C	rs699066732	*TMPRSS2*	ENSSSCT00000041631.2	missense variant	I258V	deleterious	0.02	YES
13:204887898	G/T	rs711589152	*TMPRSS2*	ENSSSCT00000041631.2	missense variant	S209R	tolerated	0.09	NO
13:204887942	A/T	rs703753915	*TMPRSS2*	ENSSSCT00000041631.2	missense variant	F195I	deleterious	0.02	YES
13:204895495	T/C	rs337083256	*TMPRSS2*	ENSSSCT00000041631.2	missense variant	I97V	tolerated	1.00	NO
13:204901994	T/C	rs793686550	*TMPRSS2*	ENSSSCT00000041631.2	missense variant	Q52R	tolerated	0.09	NO

References

Afar, D.E., Vivanco, I., Hubert, R.S., *et al.* (2001) Catalytic cleavage of the androgen-regulated TMPRSS2 protease results in its secretion by prostate and prostate cancer epithelia. Cancer Res. 61, 1686-1692. https://cancerres.aacrjournals.org/content/61/4/1686.long.

Benetti, E., Tita, R., Spiga, O., *et al.* (2020) ACE2 gene variants may underlie interindividual variability and susceptibility to COVID-19 in the Italian population. Eur. J. Hum. Genet. 1-13. https://doi.org/10.1038/s41431-020-0691-z.

Cao, Y., Li, L., Feng, Z., *et al.* (2020) Comparative genetic analysis of the novel coronavirus (2019-nCoV/SARS-CoV-2) receptor ACE2 in different populations. Cell Discov. 6, 11. https://doi.org/10.1038/s41421-020-0147-1.

Chen, R., Jiang, X., Sun, D., *et al.* (2009) Glycoproteomics analysis of human liver tissue by combination of multiple enzyme digestion and hydrazide chemistry. J. Proteome Res. 8, 651-661. https://doi.org/10.1021/pr8008012.

Chen, R., Jiang, X., Sun, D., *et al.* (2009) Glycoproteomics analysis of human liver tissue by combination of multiple enzyme digestion and hydrazide chemistry. J. Proteome Res. 8, 651-661. https://doi.org/10.1021/pr8008012.

Damas, J., Hughes, G.M., Keough, K.C., *et al.* (2020) Broad Host Range of SARS-CoV-2 Predicted by Comparative and Structural Analysis of ACE2 in Vertebrates. *bioRxiv*, https://doi.org/10.1101/2020.04.16.045302.

de Wit, E., Feldmann, F., Horne, E., *et al.* (2017) Domestic pig unlikely reservoir for MERS-CoV. Emerg. Infect. Dis. 23, 985-988. https://doi.org/10.3201/eid2306.170096.

Hiramatsu, H., Kyono, K., Higashiyama, Y., *et al.* (2003) The structure and function of human dipeptidyl peptidase IV, possessing a unique eight-bladed β-propeller fold. Biochem. Biophys. Res. Commun. 302, 849-854. https://doi.org/10.1016/s0006-291x(03)00258-4.

Hussain, M., Jabeen, N., Amanullah, A., Baig, A.A., Aziz, B., Shabbir, S., Raza, F. (2020) Structural Basis of SARS-CoV-2 Spike Protein Priming by TMPRSS2. *bioRxiv*. https://doi.org/10.1101/2020.04.21.052639.

Kirby, M., Yu, D.M., O'connor, S., Gorrell, M.D. (2010) Inhibitor selectivity in the clinical application of dipeptidyl peptidase-4 inhibition. Clin. Sci. 118, 31-41. https://doi.org/10.1042/CS20090047.

Kolb, A.F., Maile, J., Heister, A., Siddell, S.G. (1996) Characterization of functional domains in the human coronavirus HCV 229E receptor. J. Gen. Virol. 77, 2515-2521. https://doi.org/10.1099/0022-1317-77-10-2515.

Kristiansen, T.Z., Bunkenborg, J., Gronborg, M., *et al.* (2004) A proteomic analysis of human bile. Mol. Cell. Proteomics 3, 715-728. https://doi.org/10.1074/mcp.M400015-MCP200.

Lan, J., Ge, J., Yu, J., *et al.* (2020) Structure of the SARS-CoV-2 spike receptor-binding domain bound to the ACE2 receptor. Nature 581, 215-220. https://doi.org/10.1038/s41586-020-2180-5.

Li, F., Li, W., Farzan, M., Harrison, S.C. (2005). Structure of SARS coronavirus spike receptor-binding domain complexed with receptor. Science 309, 1864-1868. https://doi.org/10.1126/science.1116480.

Li, W., Zhang, C., Sui, J., *et al.* (2005). Receptor and viral determinants of SARS-coronavirus adaptation to human ACE2. EMBO J. 24, 1634-1643. https://doi.org/10.1038/sj.emboj.7600640.

Li, Z., Tomlinson, A.C., Wong, A.H., *et al.* (2019) The human coronavirus HCoV-229E S-protein structure and receptor binding. Elife 8, e51230. https://doi.org/10.7554/eLife.51230.

Luan, J., Jin, X., Lu, Y., Zhang, L. (2020) SARS-CoV-2 spike protein favors ACE2 from Bovidae and Cricetidae. J. Med. Virol., https://doi.org/10.1002/jmv.25817.

Meng, W., Brigance, R.P., Chao, H.J., *et al.* (2010) Discovery of 6-(Aminomethyl)-5-(2, 4-dichlorophenyl)-7-methylimidazo [1, 2-a] pyrimidine-2-carboxamides as Potent, Selective Dipeptidyl Peptidase-4 (DPP4) Inhibitors. J. Med. Chem. 53, 5620-5628. https://doi.org/10.1021/jm100634a.

Rasmussen, H.B., Branner, S., Wiberg, F.C., Wagtmann, N. (2003) Crystal structure of human dipeptidyl peptidase IV/CD26 in complex with a substrate analog. Nat. Struct. Biol. 10, 19-25. https://doi.org/10.1038/nsb882.

Shang, J., Ye, G., Shi, K., *et al.* (2020) Structural basis of receptor recognition by SARS-CoV-2. Nature 581, 221-224. https://doi.org/10.1038/s41586-020-2179-y.

Sun, J., He, W.T., Wang, L., *et al.* (2020) COVID-19: epidemiology, evolution, and cross-disciplinary perspectives. Trends Mol. Med. 26, 483-495. https://doi.org/10.1016/j.molmed.2020.02.008

Thoma, R., Löffler, B., Stihle, M., Huber, W., Ruf, A., Hennig, M. (2003) Structural basis of proline-specific exopeptidase activity as observed in human dipeptidyl peptidase-IV. Structure 11, 947-959. https://doi.org/10.1016/s0969-2126(03)00160-6.

Towler, P., Staker, B., Prasad, S.G., *et al.* (2004) ACE2 X-ray structures reveal a large hinge-bending motion important for inhibitor binding and catalysis. J. Biol. Chem. 279, 17996-18007. https://doi.org/10.1074/jbc.M311191200.

Wang, N., Shi, X., Jiang, L., *et al.* (2013) Structure of MERS-CoV spike receptor-binding domain complexed with human receptor DPP4. Cell Res. 23, 986-993. https://doi.org/10.1038/cr.2013.92.

Wentworth, D.E., Holmes, K.V. (2001) Molecular determinants of species specificity in the coronavirus receptor aminopeptidase N (CD13): influence of N-linked glycosylation. J. Virol. 75, 9741-9752. https://doi.org/10.1128/JVI.75.20.9741-9752.2001.

Wong, A.H., Zhou, D., Rini, J.M. (2012) The X-ray crystal structure of human aminopeptidase N reveals a novel dimer and the basis for peptide processing. J. Biol. Chem. 287, 36804-36813. https://doi.org/10.1074/jbc.M112.398842.
